# Cannabis self-administration in the human laboratory: a scoping review of ad libitum studies

**DOI:** 10.1007/s00213-023-06360-4

**Published:** 2023-05-09

**Authors:** Ke Bin Xiao, Erin Grennell, Anthony Ngoy, Tony P. George, Bernard Le Foll, Christian S. Hendershot, Matthew E. Sloan

**Affiliations:** 1 Addictions Division, Centre for Addiction and Mental Health, Toronto, Ontario Canada; 2 Department of Pharmacology & Toxicology, University of Toronto, Toronto, Ontario Canada; 3 Biobehavioural Addictions and Concurrent Disorders Research Laboratory (BACDRL), Centre for Addiction and Mental Health, Toronto, Ontario Canada; 4 Department of Psychiatry, Temerty Faculty of Medicine, University of Toronto, Toronto, Ontario Canada; 5 Campbell Family Mental Health Research Institute, Centre for Addiction and Mental Health, Toronto, Ontario Canada; 6 Institute of Medical Science, University of Toronto, Toronto, Ontario Canada; 7 Translational Addiction Research Laboratory, Centre for Addiction and Mental Health, Toronto, Ontario Canada; 8 Department of Family and Community Medicine, University of Toronto, Toronto, Ontario Canada; 9 Waypoint Research Institute, Waypoint Centre for Mental Health Care, Penetanguishene, Ontario Canada; 10 Dalla Lana School of Public Health, University of Toronto, Toronto, Ontario Canada; 11 Department of Psychiatry, University of North Carolina at Chapel Hill, Chapel Hill, NC USA; 12 Bowles Center for Alcohol Studies, University of North Carolina at Chapel Hill, Chapel Hill, NC USA; 13 Department of Psychological Clinical Science, University of Toronto Scarborough, Toronto, Ontario Canada

**Keywords:** Cannabis, Cannabis smoking, Ad libitum, Free-access, Self-administration, Human laboratory, Subjective response

## Abstract

Cannabis self-administration studies may be helpful for identifying factors that influence cannabis consumption and subjective response to cannabis. Additionally, these paradigms could be useful for testing novel pharmacotherapies for cannabis use disorder. This scoping review aims to summarize the findings from existing ad libitum cannabis self-administration studies to determine what has been learned from these studies as well as their limitations. We examined studies that specifically examined cannabis smoking, focusing on subjective response and self-administration behavior (e.g., smoking topography). A systematic search was conducted using PubMed and Embase from inception to October 22, 2022. Our search strategy identified 26 studies (total *N* = 662, 79% male) that met our eligibility criteria. We found that tetrahydrocannabinol (THC) concentration significantly affected subjective response to cannabis in some but not all studies. In general, cannabis self-administration tended to be most intense at the beginning of the laboratory session and decreased in later parts of the session. There was limited data on cannabis self-administration in adults older than 55. Data on external validity and test-retest reliability were also limited. Addressing these limitations in future ad libitum cannabis self-administration studies could lead to more valid and generalizable paradigms, which in turn could be used to improve our understanding of cannabis use patterns and to help guide medication development for cannabis use disorder.

**Supplementary Information:**

The online version contains supplementary material available at 10.1007/s00213-023-06360-4.

## Introduction

Cannabis is one of the most commonly used drugs globally (Connor et al., 2021). In the past year, cannabis use among youth has increased in western nations, with prevalence rates of 18% (SAMHSA, 2021), 20% (European Monitoring Centre for Drugs and Drug Addiction, 2021), and 27% (Health Canada, 2021) in the USA, Europe, and Canada respectively. There is evidence that both rates of use and cannabis potency are rising in the USA. Between 2002 and 2020, past-year cannabis use in American adults aged 18 years and older has increased by about 8% (SAMHSA, 2021), and cannabis potency has increased since the 1980s from about 3% tetrahydrocannabinol (THC) to 12% THC in 2012 (Volkow et al., 2014). Along with this growing trend, the perception of risk from cannabis use has also decreased over the years, with fewer individuals associating harm with weekly cannabis use in 2020 (28.2%) compared to 2015 (38.7%) (SAMHSA, 2021). Long-term use of cannabis increases the risk of developing cannabis use disorder (CUD) (NIDA, 2021), and in the USA, the past-year prevalence of CUD has risen from 1.5% in 2001-2002 to 2.9% in 2012-2013 (Hasin et al., 2015).

Chronic cannabis use may increase the risk of other substance use disorders (Blanco et al., 2016) and increase the risk for and persistence of psychotic symptoms (Kuepper et al., 2011) and psychosocial impairment (Sorkhou et al., 2021). Given the adverse effects of cannabis, it is essential to gain a better understanding of how individuals self-administer the drug and its psychopharmacological effects. Although observational studies have been helpful in identifying the effects of cannabis, these studies have varying methodologies. They often rely on retrospective reports, which can be biased due to variations in the potency and type of cannabis used, co-use of other drugs, and recall bias. In contrast, cannabis self-administration (CSA) studies conducted in the laboratory allow for a potentially more valid examination of drug intake and subjective response, as researchers can control various external factors, such as cannabis potency and timing of administration. Understanding the reinforcing and subjective effects of cannabis may help us better understand who is most liable to develop CUD. Furthermore, reliable drug self-administration paradigms can be used for pharmacotherapy development (Panlilio et al., 2016; Ray et al., 2021). In order to model specific aspects of addiction, various drug self-administration designs have been developed, such as operant self-administration procedures in which participants are required to complete a task (e.g., pressing buttons) in order to receive the drugs (Haney, 2009; Stangl et al., 2022), controlled-smoking procedures (e.g., smoking inhalation guided by an experimenter’s instruction or cues) to standardize consumption (Kayser et al., 2021), choice procedures where participants are given the option to choose between the drug and one or more alternatives (e.g., money or other drugs) (Haney, 2009; Jones & Comer, 2013; McKee, 2009; Sloan et al., 2022), and free-access or ad libitum procedures (Gowin et al., 2017; Sloan et al., 2020). Ad libitum procedures are one of the most common self-administration paradigms (Chukwueke & Le Foll, 2019; Gowin et al., 2017; Jones & Comer, 2013; Sloan et al., 2020). In this type of study design, participants can freely administer a drug without restriction, although certain ceilings are often imposed for safety or practical reasons. In the present study, we specifically focused on ad libitum cannabis self-administration studies. Other forms of cannabis administration may also be reflective of real-world use (e.g., paradigms where participants pay for access to cannabis) but were beyond the scope of the current review.

The psychoactive effects of cannabis are derived from delta-9-tetrahydrocannabinol (THC). THC is rapidly absorbed into the bloodstream and peaks shortly after administration, usually in about 3-10 min when administered through inhalation (Grotenhermen, 2003). THC then acts as a partial agonist on cannabinoid receptor 1 (CB1) and cannabinoid receptor 2 (CB2) in the brain, with the psychoactive effects mediated by CB1 (Pacher et al., 2006; Sloan et al., 2019; Zou & Kumar, 2018). Cannabis can be administered recreationally through various methods, such as smoking, vaping, or oral ingestion. Although some studies have examined the effects of vaping (Spindle et al., 2019) and oral administration (Fogel et al., 2017), cannabis smoking remains the most common form of administration in both real-world (Health Canada, 2021) and laboratory settings (Russell et al., 2018; Vinette et al., 2022), so our review will focus on this administration method.

The present scoping review aims to summarize findings from ad libitum paradigms that have been used to study cannabis self-administration in the human laboratory to date. We will first summarize the design of ad libitum CSA studies and their subjective and behavioral findings. We will then discuss the test-retest reliability and external validity of these studies by reporting on correlations between repeated sessions and associations between cannabis use in the lab and external cannabis use. Finally, we will discuss the gaps in the literature and considerations for the design of future CSA studies.

## Methods

Free-access or ad libitum paradigms are one of the most straightforward methods of measuring self-administration behavior. They are thought to be reflective of real-world use, as participants can consume the drug as desired. There are usually some restrictions such as a fixed timeframe or a maximum quantity of drug consumption allowed (i.e., ceiling) to ensure practicality and participant safety.

Included studies needed to be published in English and must have employed an ad libitum human laboratory cannabis self-administration paradigm with adult participants aged 18 years and older. In our review, we only included laboratory paradigms that allowed participants to smoke cannabis for at least 10 min. This minimum time window was selected to give sufficient time for participants to reach peak intoxication state (Grotenhermen, 2003). No CSA studies were found that were less than 10 min in duration. Studies must also have included information about either subjective response to cannabis or self-administration behavior (i.e., smoking topography or amount consumed). Studies that included co-administration of alcohol, other drugs, or other forms of cannabis (e.g., oral cannabis) were excluded as we aimed to specifically investigate studies probing the reinforcing effects of smoked cannabis in the absence of other drugs. Studies in which active drug or placebo were administered prior to cannabis self-administration were also excluded due to the possibility that the active drug or placebo would influence subjective response or self-administration behavior. CSA studies that looked at other forms of cannabis administration (e.g., oral administration, vaporization) or that only used other types of paradigms (e.g., controlled-smoking procedures) were also excluded. Studies in which the cannabis was self-supplied (e.g., mobile laboratory, local dispensaries) were excluded due to the lack of standardization in THC concentration. Studies with outcomes that were not related to either subjective response or CSA behavior, such as pharmacokinetic information or device sensitivity, were excluded.

The present scoping review was initially performed using PubMed from inception to March 7, 2021. An additional literature search was conducted on October 22, 2022 to include Embase. Relevant articles were determined from searching the title and abstract using the following keywords: “marijuana smoking,” “cannabis,” “self-administration,” “free-access,” and “ad libitum”. We also added the keywords “validity,” “reliability” and “reproducibility” after the final search strategy to see if we were able to capture any specific papers about external validity and test-retest reliability in ad libitum CSA studies. For additional information about the search strategy, see Supplementary Table 1.1 and 1.2. Additional articles were identified by checking citations in included papers. Articles were selected by two independent reviewers (initial search: KX and EG, second search: KX and AN). Abstracts were screened against the eligibility criteria using Covidence (Veritas Health Innovation, 2021), a web-based software. Duplicate papers were removed by Covidence. Discrepancies in study selection were resolved by a third author (MS). The data extraction was conducted by one author (KX) and verified by another author (EG for the initial extraction, AN and MS for the second extraction).

**Fig. 1:**
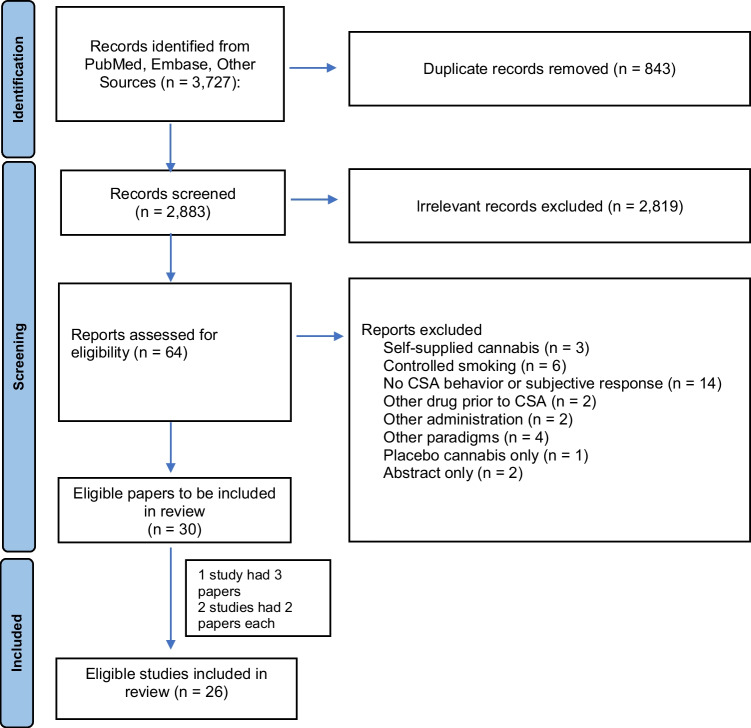
Study selection flow diagram

Our updated search identified 3727 papers, and based on title and abstract screening, 64 articles were selected. During the full-text screening, 34 papers were excluded; see details in Fig. 1 and Supplementary Table 2. For articles that consisted of only an abstract (e.g., conference papers) or that did not specify the type of self-administration used (e.g., ad libitum or controlled-smoking procedure), additional information was obtained by emailing the authors when possible.

Data extracted from the eligible papers included study design, sample size, mean age of the sample, the study’s inclusion criteria for baseline cannabis use, cannabis potency, requirements for abstinence prior to the CSA session, instructions to participants, and the duration of the ad libitum session. We also extracted outcome data related to subjective response and CSA behavior (i.e., smoking topography outcomes and amount of cannabis administered). Additional data were included if they were thought to be related to subjective response or CSA behavior. The study characteristics and results can be found in Table 1. Other Sources = articles from the preliminary search and in-text citations.

**Table 1:** Study characteristics and results

Study	Sample size	Participant details	Sex	Age mean ± SD	Study objectives	Study design	THC potency	Subjective response tools and data collection intervals	Subjective response results	Time of peak subjective response	Behavioral measurement results (e.g., amount consumed, smoking topography variables)	Other outcome measurement and results
Brands et al. (2019); Matheson, Mann et al. (2020); Matheson, Sproule et al. (2020)	^a^ *N* = 91	Cannabis use inclusion criteria: 1-4 days per week, tested positive for THC on urine drug screen and does not meet DSM-IV criteria for lifetime cannabis dependence Baseline cannabis use: varied between groups, ranging from 2.4 to 2.8 days per week	71% male	22 ± 2.00**	“…to examine the acute and residual (24 and 48 h after smoking) effects of smoked cannabis on simulated driving measures (driving speed, lateral control), self-reported drug effects and heart rate in young adults aged 19-25 years” (Brands et al., 2019) “To examine acute and residual mood and cognitive performance…We sought to determine if the use of a higher THC content cigarette would show more consistent effects of smoked cannabis on measures of mood and cognitive performance.” (Matheson, Mann, et al., 2020) “…to examine sex differences in THC pharmacokinetics and in acute subjective, physiological, and cognitive effects of smoked cannabis in a sample of regular cannabis users” (Matheson, Sproule, et al., 2020)	Parallel study design with 1 CSA session. Participants were randomized to receive active cannabis (12.5% THC) or placebo (0.009% THC) using a 2:1 allocation ratio. Participants and staff were blinded to potency. All participants were given a single cannabis cigarette (750 mg). Ad libitum procedure: participants were given 10 min to smoke to their desired “high”. Hours of abstinence before session: 48 h. Abstinence verification: urine drug screen.	0.0% and 12.5%	7-item VAS collected at baseline, 5, 15, 30, 60, 120, 180, 240, 300, and 360 min after CSA ARCI short form and POMS collected at baseline and 60 min after CSA	Ratings for VAS “drug effect” and “drug high” scores were significantly higher in the active cannabis group than the placebo group. Ratings for “liking” and “feels like cannabis” in females were significantly lower than in males from 180 to 360 min after CSA. Despite having similar subjective peak time, scores for females returned to baseline levels sooner than males. Females had fewer correlations between THC *C*max and the number of VAS peak effects (2/7 effects) than males (6/7 effects). Significant group effects were found for 8 subscales of POMS at 60 min (“Tension-Anxiety,” “Friendliness,” “Fatigue,” “Confusion,” “Vigor,” “Elation,” “Arousal” and “Positive Mood”). Ratings for “Friendliness” and “Elation” remain elevated in the high blood THC group at 60 min and 24 h as compared to the placebo group.	5 min post CSA (High)	Amount smoked: estimated dose of THC (calculated from weight of cigarette post-smoking) in the active cannabis group was lower in females than males. Smoking topography: similar smoking duration between males and females. Active cannabis group smoked longer than the placebo group. 7.0 ± 2.1 min for participants with high blood THC concentration, 6.0 ± 2.0 min for participants with low blood THC concentration, and 5.7 ± 1.6 min for participants in the placebo group. However, the only significant difference was between the high blood THC group and placebo group.	Driving outcomes (i.e., speed, and lateral control), whole blood THC concentration, heart rate, blood pressure, cognitive performance. Mean THC concentrations were highest at 5 min post-smoking. THC levels were higher in males than females.
Cappell et al. (1973)	*N* = 12	Cannabis use inclusion criteria: ND Baseline cannabis use: an average of twice per week	100% male	ND Age Range: 21-28	“In summary, there is an appreciable amount of evidence to suggest that nonpharmacological variables may exert control over the subjective response to marihuana in ‘socially relevant’ (i.e., low) doses. The present research was designed to explore this issue further in the context of an *ad lib* self-administration experiment.”	Sequential design with 4 CSA sessions (separated by weekly intervals), using 3 cannabis potencies (0.2%, 0.4%, and 0.8% THC). In session 2, participants were given 0.4% THC which served as a reference point for subsequent sessions. Sessions 3-5 were counterbalanced. CSA behavior was monitored and recorded by study staff. Participants and staff were blinded to cannabis potency. Ad libitum procedure: cannabis cigarettes were given to participants upon request. Participants were asked to smoke until their “optimal high,” and could end the CSA early if necessary. Hours of abstinence before session: ≥ 24 h Abstinence verification: ND	0.2%, 0.4%, and 0.8%	Verbal rating of cannabis potency following the end of the CSA.	Subjective response was not directly measured. Ratings of cigarette potency increased with THC concentration (*p* <0.01). Cigarettes with 0.2%, 0.4%, and 0.8% THC had mean ratings of 44.50, 58.16, and 66.40, respectively.	ND	Amount consumed: cannabis potency had a significant inverse relationship with amount (grams) of cannabis consumed. 0.2% and 0.4% THC were consumed 36.9% and 8.2% more than 0.8% THC, respectively. Consumption of 0.2% THC was also greater than 0.4% THC by 25.6%. Smoking topography: there was no statistically significant difference between number of puffs by condition (although the number of puffs was numerically higher for the 0.2% condition vs. the 0.4% and 0.8% condition). Mean puff duration and interpuff interval were not related to potency.	Heart rate, blood pressure, conjunctival injection, behavioral and physiological data
Chait (1989)	*N* = 10	Cannabis use inclusion criteria: healthy (based on judgment) with no history of substance use disorder according to the DSM-III criteria (exception: tobacco dependence) Baseline cannabis use: ranged from 1 to 6 days/week	80% male	23 ± ND	“The role of marijuana delta-9-tetrahydrocannabinol (THC) content in controlling marijuana smoking behavior was examined”	Cross-over design with 15 CSA sessions (and one practice CSA session with medium potency cannabis that was not included in analyses). Sessions were held twice per week (usually on Mondays and Thursdays) using three cannabis potencies (5 sessions per THC potency). Order of CSA sessions was randomized and counterbalanced. Participants and staff were blinded to potency. Ad libitum procedure: participants were given 30 min to smoke multiple half-length cannabis cigarettes; however, CSA was terminated early if the participant informed the experimenter that they wanted to stop. Cigarettes were handed to participants by staff, one at a time, as requested. Hours of abstinence before session: 24 h. Abstinence verification: ND.	^b^ 0.9%, 1.7%, and 2.7%	VAS, POMS, ARCI collected at 5, 20, and 60 min after CSA	“High” and “stimulated” ratings were similar for 1.7% THC and 2.1% THC. Both 1.7% and 2.1% THC ratings were higher than 0.5% THC.	5 min post CSA (high)	Amount consumed: there was no difference in cigarette number between the different cannabis potencies. A mean of 3.1 half-length cigarettes were smoked. The mean cutoff time (time when participants signaled, they wanted to stop smoking) was 20 min across all conditions. The cutoff time was associated with the number of cannabis cigarettes used (*r* = 0.794). Smoking topography: N/A	Heart rate, carbon monoxide level. Heart rate peaked 5 min post-smoking and decreased afterwards
* Heishman et al. (1989)	*N* = 12	Cannabis use inclusion criteria: ND Baseline cannabis use: all participants reported prior history of cannabis use, but 10 participants reported using an average of 7.8 times per month and an average of 2.1 joints per occasion	100% male	31 ± 5.8	“…to examine smoking behavior across multiple doses of marijuana in order to characterize potential behavioral adjustments.”	Cross-over design with 3 CSA sessions (spaced apart by at least 48 h) using 3 cannabis potencies (0%, 1.3%, and 2.7% THC). CSA sessions were held between 9AM and 11AM and order of potencies were counterbalanced. Ad libitum procedure: participants were given a single cannabis cigarette and were forced to stop smoking after the eighth puff. Participants were given only standardized instruction for puff initiation, but other than that, participants could smoke each of the 8 puffs however they liked. ND on time. Hours of abstinence before session: 48 h Abstinence verification: urine drug screen	0%, 1.3%, and 2.7%	9-items on a VAS rated from 0 to 100. Subjective report assessment was collected at 5, 25, 45, and 65 min post CSA	Drug “high”, “stoned”, and “impaired performance” ratings had significant dose effects, with participants reporting greater “high” and “impaired performance” ratings on active doses of cannabis (1.3% and 2.7%) than on the placebo dose. “Clear-headed” rating was significantly lower under active cannabis condition relative to placebo.	5 min post CSA (high)	Amount consumed: N/A Smoking topography: no significant difference was found between 1.3 and 2.7% THC in smoking duration (min). Puff duration and puff volume were lower under the 2.7% THC condition versus the 1.3% THC condition. No difference was found between the active and placebo cannabis in interpuff interval, maximum flow rate, and average flow rate.	Heart rate, cognitive/psychomotor task performance
* Herning et al. (1986)	*N* = 10	Cannabis use inclusion criteria: ND Baseline cannabis use: an average of 72 (± 65) times per month	100% male	29 ± 6	“We compared marijuana cigarettes with over a three-fold difference in THC content. Thus, in this study we maximized the chances of finding evidence for dose regulation.”	Cross-over design with 2 CSA sessions (separate days) using two cannabis potencies; 1.2% THC and 3.9% THC. Order of cannabis self-administration sessions was counterbalanced. Participants and staff were blinded to potency. Ad libitum procedure: participants were given 1 cannabis cigarette (about 900 mg) per CSA session. Cigarette had to be smoked down to an experimenter-specified length. ND on time. Hours of abstinence before session: ND. Abstinence verification: ND.	1.2% and 3.9%	Verbal self-report intoxication rating (0-100) collected three times before smoking or until stable values were collected and at 0, 5, 10, 15, 20, 30, 45, 60, 90, and 120 min after the last puff	“High” rating for 3.9% THC cannabis (65.80 ± 29.66) was significantly higher than 1.2% THC cannabis (48.20 ± 28.17).	15 min post CSA (high)	Amount consumed: N/A Smoking topography: more puffs, longer interpuff intervals, and larger inhalation volume were taken with 3.9% THC cannabis than 1.2% THC cannabis. No significant differences in average puff volume, puff duration, and inhalation duration. Participants took approximately twice as long to smoke the 3.9% THC than 1.2% THC. Greater cumulative puff volume and inhalation volume were found with 3.9% THC cannabis.	Heart rate, blood pressure, skin temperature, expired carbon monoxide
Herrmann et al. (2015)	*N* = 7	Cannabis use inclusion criteria: “using cannabis at least two times per week throughout the past 90 days, and provided a urine specimen that was positive for THCCOOH,” and did not meet current past-year substance use disorder Baseline cannabis use: ND	57% male	29 ± 5.8	“This report examines the physiological, subjective, and behavior/cognitive effects of secondhand cannabis exposure, and the influence of room ventilation on these effects.”	Before-and-after design with 3 CSA sessions using 2 cannabis potencies in both unventilated and ventilated rooms. The 3 CSA sessions were (i) 5.3% THC in an unventilated environment, (ii) 11.3% THC in an unventilated environment, and (iii) 11.3% THC in a ventilated environment. However, results were only reported for the 11.3% THC sessions in this manuscript. Ad libitum procedure: participants were given 10 cannabis cigarettes (about 1 g each) and 60 min to smoke. Participants smoked simultaneously in the room. Hours of abstinence before session: participants were instructed to remain abstinent from cannabis overnight prior to CSA sessions. Abstinence verification: study staff met with participants at start of session to check for signs of recent cannabis use (e.g., blood shot eyes, odor of cannabis smoke).	11.3% ^c^	15-item DEQ collected at baseline, 0, 0.5, 1, 1.5, 2, 3, 4, 6, and 8 h post exposure	Subjective response in this study was first measured after the 60-min CSA was completed. Ratings of “drug effect,” “pleasant drug effect,” “hungry/have munchies,” “relaxed,” and “vigorous” were higher than baseline following CSA but decreased to baseline level within 4 h.	N/A	Amount consumed: an average of 2.6 g (± 0.5) was smoked each session. Participants smoked more cannabis in the ventilated session (16.5 g total) than the unventilated session (14.4 g total), but the authors did not address whether this was a statistically significant difference. Smoking topography: based on puff volume, cannabis smokers consumed 34% less cannabis in the second half of the hour than the first half.	THC level (whole blood), heart rate, blood pressure, behavioral/cognitive performance
Hoffman et al. (2021); Marcotte et al. (2022)	^d^ *N* = 191 Frequent cannabis users (*N* = 93) Occasional cannabis users (*N* = 98)	Cannabis use inclusion criteria: ≥ 4 times per month, Baseline cannabis use: 16.7 (± 9.8) days in last 30 days “No participants met criteria for cannabis use disorder”. “Participants were stratified into frequent (≥ 4 times per week) or occasional users (<4 times per week)”. An analysis stratified participants into 3 subgroups based on cannabis use intensity in the past 6 months using the Timeline Follow-back interview (Marcotte et al., 2022).	62% male	30 ± 8.3	“We aim to characterize blood and [oral fluid] concentrations up to 6 h post-cannabis smoking in frequent and occasional users and to determine detection windows using current drug *per se* limits.” (Hoffman et al., 2021) “To determine, in a large sample of regular cannabis users, the magnitude and time course of driving impairment produced by smoked cannabis of different ∆9-tetrahydrocannabinol (THC) content, the effects of use history, and concordance between perceived impairment and observed performance.” (Marcotte et al., 2022)	Parallel, double blinded, between-subject study design with 1 CSA session. Participants were stratified into frequent and occasional cannabis users and randomized to placebo cannabis or one of two active cannabis conditions (5.9% or 13.4% THC). Ad libitum procedure: participants were given 10 min to smoke a single cigarette (700 mg). At least 4 puffs were required. Hours of abstinence before session: 48 h. Abstinence verification: each participant’s oral fluid THC concentration as tested using a Draeger 5000 and if THC concentration was ≥5 ng/mL, they were excluded. Result was later confirmed by liquid chromatography tandem mass spectrometry, which led to 7 additional participants being excluded from analyses.	0%, 5.9%, 13.4%	0-100 “high” rating collected at 15 min after CSA	The 5.9% THC group reported significantly greater subjective “high” compared to the 13.4% THC group (median of 71 and 55.5, respectively). There was no correlation between amount of cannabis smoked based on weight and self-reported subjective response. There was no difference in subjective “high” reports between frequent and occasional users.	ND	Amount consumed: there were no differences in the amount of cigarette (grams) smoked between the 0%, 5.9%, and 13.4% THC groups. In the 5.9% THC group, frequent users smoked more of the cigarette (0.53 g) than occasional users (0.34 g). Smoking topography: In the two active conditions (5.9% THC and 13.4% THC), individuals smoked for longer (an average of 7.2 and 7.0 min respectively) than in the placebo group (an average of 6.0 min). However, there were no differences in puff number between the three groups. In the 5.9% THC group, frequent users took more average puffs (22.2) than occasional users (15.0). No differences were found in puffs or the amount of cigarette smoked between the frequent and occasional users in the 13.4% or placebo group.	Driving score, driving performance, crashes, perception of effects, pharmacokinetic profile (i.e., whole blood and oral fluid THC). About 15 min after smoking started, a significant difference in blood THC concentration was found between the placebo, 5.9% THC and 13.4% THC groups, with the 5.9% THC group having the highest concentration. Post-smoking blood THC concentration differed significantly between the three intensity subgroups. The lowest intensity group had the lowest blood THC concentration, followed by the middle intensity group, with the highest intensity group having the highest blood THC concentration.
Matthias et al. (1997)	*N* = 10	Cannabis use inclusion criteria: ND Baseline cannabis use: an average of 12.7 (± 11.5) joints per week	100% male	23 ± 2.3	“To determine whether smoking more, compared to less, potent marijuana (MJ) cigarettes to a desired level of intoxication (‘high’) reduces pulmonary exposure to noxious smoke components”	Cross-over design with 3 CSA sessions (approximately 1 week apart) using placebo cannabis and two cannabis potencies. Order of CSA sessions was randomized and counterbalanced. Participants were blinded to the potency. Ad libitum procedure: participants were given 1 cannabis cigarette per CSA session. Participants were instructed to stop smoking once they had reached their desired “high”. ND on time. Hours of abstinence before session: ≥ 6 h. Abstinence verification: ND.	0.0%, 1.77%, and 3.95%	0-10 intoxication rating collected immediately before smoking and 2, 5, 15, 30, and 45 min after CSA	No statistically significant difference in “high” ratings between the different cannabis potencies, although there were numerical differences (placebo rating = 3.0/10, 1.77% THC rating = 4.3/10, 3.95% THC rating = 6.0/10).	ND	Amount consumed: N/A Smoking topography: no difference in puff numbers, average puff volume, cumulative puff volume, interpuff interval, inhaled volume, breath-holding time, or butt-length between the different cannabis potencies.	Heart rate, deposition of tar and THC, blood carboxyhemoglobin boost
McClure et al. (2012)	*N* = 20	^e^ Cannabis use inclusion criteria: “used cannabis at least 25 days/month and provided a urine specimen positive for cannabinoids… did not meet criteria for primary sleep or Axis-1 psychiatric disorders (DSM-IV) other than nicotine or cannabis dependence; 6) were not seeking treatment for cannabis-related problems or using cannabis for a medical disorder, 7) had a negative urine toxicology test for drugs other than cannabis…” Baseline cannabis use: an average of 4 (± 3) times per day 50% of the participants met DSM-IV criteria for either cannabis abuse or cannabis dependence	85% male	29 ± 8	“…to measure cannabis smoking topography characteristics during periods of ad libitum use and to correlate topography assessments with measures of self-reported cannabis use, withdrawal and craving during abstinence, and cognitive task performance.” ^e^ “Specific aims of the study were to 1) examine the effects of cannabis withdrawal on sleep continuity and architecture, and 2) determine whether administration of extended-release zolpidem…could attenuate withdrawal-induced sleep disturbance.”	Cross-over design with participants completing two inpatient admissions. During each inpatient admission, participants completed two consecutive days of CSA sessions using a single cannabis potency from 12 to 9 PM each day, followed by a 3-day period of supervised cannabis abstinence. Prior to each 3-day period of abstinence, participants received either 12.5 mg of extended-release zolpidem or placebo in a counterbalanced order. The two inpatient admissions were separated by a 1-week outpatient washout period. Ad libitum procedure: cannabis cigarettes were given by the study nurse upon request. Hours of abstinence before session: ND. Abstinence verification: ND.	3%	ND	ND	ND	Amount consumed: a mean of 12 (± 5) cigarettes was smoked during each CSA day. Smoking topography: there were no significant differences in smoking topography measures between the CSA periods. Smoking topography values (i.e., total volume, total volume per cigarette, average puff volume, and puff velocity) on day 2 of CSA period were consistently higher than day 1, suggesting that participants smoked more on the days before the cannabis abstinence period. Smoking intensity decreased with progressive puffs. Puff volume and duration gradually decreased over the course of each cigarette being smoked. Puff volume, puff duration, and time to peak velocity were significantly higher for the first four puffs of the cigarette than the last four puffs (for the first and last cigarette of each day). Years of frequent cannabis use were positively associated with total volume per cigarette, puff volume, puff duration, and puff velocity. Total puff volume and maximum puff duration were positively correlated with the number of times cannabis was used per day in the last 30 days.	Cannabis withdrawal, cannabis craving, sleep quality, cognitive performance
* Meyer et al. (1971)	*N* = 12 Heavy users (*N* = 6) Casual users (*N* = 6)	Cannabis use inclusion criteria: ND Baseline cannabis use: heavy users used cannabis almost daily. Casual users used cannabis once a week or less	^f^ 100% male	ND	“Physiologic measures, performance measures, and subjective effects were recorded in order both to differentiate the two groups of subjects in the marijuana condition and to compare the groups on a placebo.”	Cross-over study design with 3 CSA sessions (separated by weekly intervals) using 3 potencies (250 mg of marijuana leaf, placebo cannabis, and self-selected amount of marijuana leaf). Participants and staff were blinded when the fixed dose of cannabis or placebo cannabis was administered. The CSA sessions were counterbalanced. Ad libitum procedure: participants were given 30 min to smoke the cannabis in the pipe provided. During the placebo and fixed dose sessions, participants were required to finish the dose before refilling it. During the self-selected dose session, participants were asked to smoke to their usual “high” Hours of abstinence before session: ND Abstinence verification: ND	0.9% (placebo condition: 0 mg) (fixed dose: 250 mg) (Ad lib dose: self-selected)	5-point scale “high” rating was collected 30 and 90 min post CSA	5/6 heavy users reported themselves to be “very high” or “extremely high” 30 min after the ad lib dose, whereas one casual user reported “very high”. The majority of casual users (4/6) reported that they felt “moderately high” 30 min after the ad lib dose. Heavy users reported greater level of “high” than casual users at 30 min post CSA, but also less “high” at 90 min post CSA compared to casual users. The authors did not report whether these differences were statistically significant. During the placebo CSA, most participants felt at least “slightly high”.	ND	Amount consumed: during the CSA session with self-selected dose, the dose ranged from 160 mg of THC to 570 mg of THC. No significant difference was found between the heavy users and casual users in ad lib dose smoked (380 mg and 420 mg, respectively). Smoking topography: N/A	Psychological task performance
* Miller and Cornett (1978)	*N* = 16	Cannabis use inclusion criteria: ND Baseline cannabis use: 2-4 times per week	100% male	ND Age range: 21-28	“Since dosage variables may have played a role in the discordant findings, dosage of THC contained in marijuana was varied in the present study to determine whether changes in memory strength as measured by d’ would occur for recognition memory following a test for free recall.”	Cross-over design with 4 CSA sessions (separated by 1 week) using 4 cannabis potencies (0, 5, 10, 15 mg of THC). Order of cannabis potencies was randomized and counterbalanced. Ad libitum procedure: participants could smoke in any manner they liked but “were instructed to finish as much of the butt as possible.” Hours of abstinence before session: ND Abstinence verification: ND	0, 5, 10, and 15 mg of THC	0-100 ratings of “potency/high” and “pleasantness” were collected at the end of the memory tests	Subjective ratings increased by potency. There were significant differences in “potency/high” and “pleasantness” between the placebo and 5 mg doses, the 5 mg and 10 mg doses, and the 10 mg and 15 mg doses.	ND	Amount consumed: N/A Smoking topography: N/A	Pulse rate, recall
* Miller, Cornett, Brightwell, McFarland, Drew et al. (1977)	*N* = 40	Cannabis use inclusion criteria: ND Baseline cannabis use: 2-4 times per week	100% male	ND Age range: 21-28	“…to determine the effect of marijuana on storage and retrieval processes in the free recall of prose material in the presence and absence of retrieval cues.”	2 CSA sessions (separated by 24 h) using active cannabis and placebo cannabis. Participants were randomized to receive either active or placebo cannabis in the first CSA session. In the second CSA session, half of the participants in the first CSA were randomly assigned to the same condition or switched to the opposite drug. Participants smoked in groups of 4. Ad libitum procedure: Participants could smoke in any manner they liked but “were instructed to finish as much of the cigarette as possible.” Hours of abstinence before session: ND Abstinence verification: ND	2.1% and placebo	0-100 ratings of “potency/high” and “pleasantness” were collected at the end of the memory tests	Users with active cannabis reported greater “potency/high” and “pleasantness” ratings than the placebo group on both CSA days.	ND	Amount consumed: N/A Smoking topography: N/A	Pulse rate, recall memory
* Miller, Cornett, Drew, McFarland, Brightwell et al. (1977)	*N* = 32	Cannabis use inclusion criteria: ND Baseline cannabis use: 2-4 times per week	100% male	ND Age range: 21-28	“…to clarify the relationship between changes in pulse rate following intoxication and subjective estimates of ‘high’ and ‘pleasantness’.”	Parallel study session with 1 CSA session where participants received either placebo cannabis or 5, 10, or 15 mg of THC. Participants smoked in groups of 8 individuals. Ad libitum procedure: participants could smoke in any manner they liked but “were instructed to finish as much of the butt as possible.” Hours of abstinence before session: 4 days Abstinence verification: ND	0, 5, 10, and 15 mg THC (ND on THC %)	0-100 ratings of “potency/high” and “pleasantness” were collected at the end of the memory tests	“Potency” and “pleasantness” ratings increased as a function of THC dosage: Placebo: potency – 21.3. Pleasantness – 43.3 5 mg: potency – 41.2. Pleasantness – 62.9 10 mg: potency – 63.8. Pleasantness – 62.5 15 mg: potency – 68.8. Pleasantness – 79.6	ND	Amount consumed: N/A Smoking topography: N/A	Pulse rate, recognition memory
* Miller et al. (1978)	*N* = 12	Cannabis use inclusion criteria: ND Baseline cannabis use: 2-4 times per week to a few times per month	100% male	ND Age range: 21-30	“…to evaluate the effect of marijuana on storage, retention and retrieval processes simultaneously”	Cross-over design with 2 CSA sessions (spaced apart by 1 week) using active cannabis and placebo cannabis. Participants were randomized to receive either active or placebo cannabis in the first CSA session, and received the other potency during the next CSA session. Ad libitum procedure: participants could smoke in any manner they liked but “were instructed to finish as much of the butt as possible.” Hours of abstinence before session: 4 days Abstinence verification: ND	2.1% and placebo	0-100 ratings of “potency/high” and “pleasantness” were collected at the end of the memory tests	Active cannabis was rated as having greater “potency/high” and “pleasantness” (63.3 and 63.9, respectively) than placebo cannabis (23.3 and 31.0, respectively).	ND	Amount consumed: N/A Smoking topography: N/A	Pulse rate, recall
* Miller et al. (1979)	*N* = 12	Cannabis use inclusion criteria: ND Baseline cannabis use: 2-4 times per week to a few times per month	100% male	ND Age range: 21-30	“…to assess the effects of marijuana on the multiple measures of memory described. Free, delayed and serial recall measures were utilized.”	Cross-over design with 2 CSA sessions (spaced apart by 1 week) using active cannabis and placebo cannabis. Participants were randomized to receive either active or placebo cannabis in the first CSA session and received the other potency during the next CSA session. Participants smoked in groups of 3 to 4 individuals. Ad libitum procedure: Participants could smoke in any manner they liked but “were instructed to finish as much of the cigarette as possible.” Hours of abstinence before session: ND Abstinence verification: ND	0 and 10 mg THC (ND on THC %)	0-100 ratings of “potency/high” and “pleasantness” were collected at the end of the memory tests	Active cannabis was rated as having greater “potency/high” and “pleasantness” (57.6 and 51.9, respectively) than placebo cannabis (39.0 and 30.9, respectively).	ND	Amount consumed: N/A Smoking topography: N/A	Pulse rate, recall
* Miller, McFarland, Cornett, and Brightwell (1977)	*N* = 34	Cannabis use inclusion criteria: ND Baseline cannabis use: 2-4 times per week	100% male	ND Age range: 21-28	“…to assess the effect of marijuana on repeated free recall of same and different word lists…to evaluate the effect of marijuana on intrusion errors…to ascertain how well information would be retained following marijuana providing initial recall had occurred.”	Parallel study design with 1 CSA session where participants were randomized to receive either active cannabis or placebo cannabis. Participants were asked to smoke 1 g cannabis cigarettes in groups of 4 or 5 individuals. Each group had a mix of active and placebo cannabis smokers. Ad libitum procedure: participants could smoke in any manner they liked but “were instructed to finish as much of the butt as possible.” Hours of abstinence before session: ND Abstinence verification: ND	1.4% and placebo	0-100 ratings of “potency” and “pleasantness” were collected at the end of the memory tests	Active cannabis smokers reported greater “potency” (56.5) and “pleasantness” (58.2) than the placebo group (18.4 and 31.6 for potency and pleasantness, respectively).	ND	Amount consumed: N/A Smoking topography: N/A	Pulse rate, recall memory, intrusions, retention memory, recognition memory
* Miller, McFarland, Cornett, Brightwell et al. (1977)	*N* = 28	Cannabis use inclusion criteria: ND Baseline cannabis use: 2-4 times per week	100% male	ND Age range: 21-28	“…the drug may have differential effects on the recall of pictures and words with recall of the latter being more impaired. One purpose of the present study was to test this hypothesis. Another purpose was to determine the role of subjective organization in free recall.”	Cross-over design with 2 CSA sessions (spaced apart by 1 week) using active cannabis and placebo cannabis. Participants received either active or placebo cannabis in the first CSA session and received the other potency during the next CSA session. Ad libitum procedure: participants could smoke in any manner they liked but “were instructed to consume as much of the cigarette as possible.” Hours of abstinence before session: 4 days Abstinence verification: ND	1.4% and placebo	0-100 ratings of “potency/high” and “pleasantness” were collected at the end of the memory tests	Active cannabis was rated more potent and pleasant (63.5 and 59.8, respectively) than placebo cannabis (rated 19.0 and 29.1, respectively).	ND	Amount consumed: N/A Smoking topography: N/A	Pulse rate, recall memory
* Perez-Reyes et al. (1981)	*N* = 6	Cannabis use inclusion criteria: ND ^CC^ Baseline cannabis use: an average of 12 cigarettes/month	50% male	^CC^ 27 ± 2.2	“…no systematic study has been made of the dynamics of marihuana cigarette smoking nor of the clinical pharmacologic effects of the sequential smoking of more than one marihuana cigarette.”	Sequential study design with 2 CSA sessions using a single potency of cannabis. The two CSA sessions were separated by 2 h. Ad libitum procedure: participants could smoke in any manner they liked but had to finish the cigarette. Hours of abstinence before session: 6 days Abstinence verification: ND	1%	“High” ratings were collected at 5, 10, 15, 20, 30, 40, 50, 60, 70, 80, 90, 100, 110, and 120 min following start of CSA Participants did not use a defined scale and were allowed to use whatever scale they liked. They were also shown their previous ratings for comparison. Participants scored their overall "high" from the CSA by comparing the “high” with the greatest “high” they had experienced; this served as a reference point.	No sex difference was found in subjective response. Feelings of “high” remained for a long time (about 3 h) despite decrease in plasma THC concentration. The overall “high” ratings were 62% for the first cigarette and 53% for the second cigarette.	30 min after start of the first CSA session and 10 min after start of second CSA session	Amount consumed: N/A Smoking topography: no significant difference in smoking duration between the two cigarettes. Between the two cigarettes, no significant differences were found in average puff number, puff duration, breath-holding duration, and time between puffs. Males consumed the cigarettes more quickly and took more puffs and took them more frequently than females, although it was not indicated whether these differences were statistically significant.	Heart rate, THC plasma concentration. The THC plasma concentration of the first cigarette was higher than the second cigarette, but the difference was not statistically significant. Females had lower area under the plasma THC vs. time curve (481 vs. 630) but the difference was not statistically significant.
* Perez-Reyes et al. (1982)	*N* = 6	Cannabis use inclusion criteria: ND Baseline cannabis use: 4 to 12 cigarettes/month	50% male	ND Age range: 26-32	“[THC] pharmacologic effects are quickly perceived, theoretically allowing the user to titrate the amount of drug inhaled to reach his or her desired level of psychologic ‘high.’ This titration could be accomplished by varying the pattern of smoking. We conducted a systematic investigation in which experienced users smoked marihuana cigarettes of three different potencies under double-blind laboratory condition to study this possibility.”	Cross-over design with 3 CSA sessions (separated by weekly intervals) using 3 cannabis potencies (1.32%, 1.97%, and 2.54% THC). Order of cannabis potencies was counterbalanced. Ad libitum procedure: participants could smoke in any manner they liked until their usual “high” or for as long as they liked. Hours of abstinence before session: 7 days Abstinence verification: ND	1.32%, 1.97%, and 2.54%	0-100 scale ratings of “high” were collected at 5, 10, 15, 20, 30, 45, 60, 75, 90, 120, 240, and 360 min following start of CSA. An overall rating was also collected 6 h post CSA. Participants were also shown their previous ratings for comparison.	Overall high rating (measured 6 h after smoking) increased by potency: 46 for 1.32% THC, 59 for 1.97% THC, and 74 for 2.54% THC. However, the difference in area under the subjective response vs. time curve for the 1.32% THC cigarette and 1.97% THC cigarette was not statistically significant.	20-30 min after start of CSA	Amount consumed: average amount of cigarette consumed was similar between the different potencies (732 mg for 1.32% THC, 627 mg for 1.97% THC, and 626 mg for 2.54% THC). Smoking topography: little difference was found in smoking topography variables (i.e., smoking time, puff number, puff duration, breath-holding time, interval between puffs, total puff time, and total breath-holding time) between the different potencies. No difference in smoking patterns was found between males and females.	Heart rate, THC plasma concentration. Plasma THC concentration peaked at 7-8 min after start of CSA and decreased afterwards, despite participants continuing to smoke. The 2.54% THC cigarette led to the highest THC plasma concentration as compared to the other dose. Females were found to have a higher plasma THC concentration than males, although it was not indicated whether this was a statistically significant difference.
* Schaefer et al. (1977)	*N* = 12	Cannabis use inclusion criteria: ND Baseline cannabis use: had prior history of cannabis use “which varied from occasional to habitual use”	100% male	ND ± 1.6 Age range: 21-38	“This reports describes the behavioral phase of a preliminary investigation into the urinary excretion of metabolites of delta-9-tetrahydrocannabinol (THC) after marihuana smoking.”	Residential study design with 3 consecutive days of CSA sessions over a 5-day admission to a research ward. On each CSA days, participants were given a single cigarette of one of three potencies; placebo, 1.5% THC or 2.2% THC. The order of the dose was counterbalanced and administered about 4 h after breakfast. On the fifth day, all participants received 2.2% THC. Ad libitum procedure: participants were asked to completely finish the cannabis cigarette using their own smoking manner. Hours of abstinence before session: ND Abstinence verification: ND	1.5% and 2.2% (placebo condition – no cannabinoids), 10 mg THC (1.5%), 20 mg THC (2.2%)	ARCI was collected 30 min post CSA. 0-10 rating score on “high” was collected before and after CSA. Participants were also given a graph to keep track of their “high” rating.	“High” rating increased significantly with cannabis potency. Placebo rating = 5.0, 10 mg THC rating = 6.9, and 20 mg THC rating = 9.3.	ND	Amount consumed: N/A Smoking topography: smoking duration typically lasted 10-15 min.	Heart rate, complex reaction time, perceptual accuracy
Schwope et al. (2012)	*N* = 10	Cannabis use inclusion criteria: “cannabis use at least twice monthly for three months before study entry.” Participants also needed to have a positive urine cannabinoid test to be eligible. Baseline cannabis use: an average of 4.9 (± 3.2) joints per day	90% male	31 ± 8.9	“The present study examined relationships between whole blood cannabinoid concentrations and pharmacodynamics effects in heavy, chronic cannabis smokers.”	Before-and-after design with 1 CSA session using a single cannabis potency. Ad libitum procedure: participants were given 10 min to smoke 1 cannabis cigarette (about 800 mg). Hours of abstinence before session: Participants were admitted to a secure research unit 15-20 h before self-administration. No restriction on cannabis use was required before study admission	6.8%	VAS collected before smoking (−0.5 h), and 0.25, 0.5, 1, 2, 3, 4, and 6 h after the start of CSA	Significant increase in “high,” “stimulated,” “stoned,” “sedated,” and “good drug effects” ratings after smoking. No significant change in “anxious” or “restless” ratings. “High” ratings returned to baseline 6 h after smoking.	15 min post CSA	ND	Cardiovascular measures, cannabis influence factor, impairment assessments, critical tracking task, whole blood cannabinoids. No data on consumption was reported but peak THC blood concentration occurred 15 min after CSA.
* Spindle et al. (2018, 2019)	*N* = 17	Cannabis use inclusion criteria: had used cannabis before but not in the past month and had a negative urine drug test at the screening visit and before the experimental sessions. Baseline cannabis use: an average of 398 (± 437) days since last cannabis use	53% male	27 ± 5.7	“To evaluate the acute dose effects of smoked and vaporized cannabis using controlled administration methods.” (Spindle et al., 2018) “This study compared the concentrations of cannabinoids in whole blood and oral fluid after administration of smoked and vaporized cannabis” (Spindle et al., 2019)	Cross-over design with 6 CSA sessions, using 3 cannabis potencies, with vaporized cannabis inhalation for 3 consecutive sessions and smoking for 3 consecutive sessions. THC dose order was randomized. Each experimental session was spaced apart by at least a week. Participants and study staff were double blinded to the dose only. Ad libitum procedure: participants were given 10 min to finish smoking cannabis in a hand-held pipe. Hours of abstinence before session: ND. Abstinence verification: Urine drug screen.	0% and 13.4% 25 mg condition (13.4% THC) 10 mg condition (mix of 13.4% THC and 0% THC) Placebo (0% THC - 0 mg condition)	DEQ collected at baseline, 10, 30, 60, 90, 120, 180, 240, 300, 360, and 480 min after CSA	Mean ratings of “drug effects,” “pleasant,” “sleepy,” and “hungry or had the munchies” were significantly higher in the 10 mg and 25 mg of THC conditions than the placebo condition for both inhalation methods. Most drug effect ratings were lower in smoked cannabis than vaporized cannabis conditions. “Drug effect” ratings remained elevated at 6 h post CSA for both 25 mg smoked and vaporized conditions. Significant correlations were found between “drug effect” and whole blood THC, 11-OH-THC, and THCCOOH and oral fluid THC after active dose administration. Correlation was highest with THCCOOH in blood.	10-30 min post CSA	ND	Cognitive/psychomotor assessments, vital signs, and cardiovascular effects, whole blood and oral fluid cannabinoid concentration. Whole blood concentration of THC, 11-OH-THC, and THCCOOH peaked within the first 10-30 min after the end of CSA for both inhalation methods. Oral fluid THC also peaked within 10 min after the end of CSA. Whole blood maximum concentrations of THC and 11-OH-THC were higher in females than males, regardless of route of administration (smoking vs. vaporized inhalation).
* Spindle et al. (2021)	^g^ *N* = 14	Cannabis use inclusion criteria: females, used cannabis “≥ 25 days per month in the past 3 months; positive urine specimen for cannabis; meet DSM-V criteria for moderate or severe CUD; and report ≥2 cannabis withdrawal symptoms during a past cannabis abstinence period” Baseline cannabis use: 83.6 (± 10.4) days of use in past 90 days, used an average of 4.8 (± 3.0) g of cannabis per day	0%	23 ± 2.7	“…we explored the relation between acute cannabis effects and mood/craving/withdrawal and CB_1_ receptor availability.”	Sequential design with 2 CSA session using 2 cannabis potencies. After the second CSA session, participants were monitored for a 3-day period of cannabis abstinence. Sessions were completed in a fixed order with placebo cannabis first followed by active cannabis. Participants and study staff were double blinded. Ad libitum procedure: participants were given 10 min to finish smoking cannabis in a hand-held pipe. Hours of abstinence before session: ND. Abstinence verification: ND.	0% and 13.4% 25 mg condition (13.4% THC) Placebo (0% THC - 0 mg condition)	21-item DEQ collected at baseline, 0, 15, 30, 45, 60, 90, 120, and 180 min after CSA	Higher ratings for “drug effect,” “pleasant,” and “like” were reported for active cannabis compared to placebo cannabis.	0-15 min post CSA	ND	Cognitive/psychomotor assessments, vital signs, withdrawal, CB1 receptor distribution, Marijuana Craving Questionnaire-Short Form, Marijuana Withdrawal Checklist
Tashkin et al. (1976)	*N* = 28	Cannabis use inclusion criteria: ND Baseline cannabis use: at least 4 days per week	100% male	24 ± ND	“To evaluate the subacute effects of heavy marihuana smoking on the lung”	Residential study with participants admitted for 94 days to a closed research unit. The first 11 days were a period of forced abstinence in which participants were not allowed to use cannabis. During the next 80 days, they were allowed to use cannabis (2.2% THC) ad libitum, except for a 1-week period from day 76 to 82. Ad libitum procedure: participants were allowed to use cannabis ad libitum over a period of 80 days (except for days 76-82). Period of abstinence before sessions: 11 days. Abstinence verification: inpatient detoxification.	2.2%	ND	ND	ND	Amount consumed: an average of 5.2 cannabis cigarettes were consumed per day. Smoking topography: N/A	Heart rate, pulmonary function
Wu et al. (1988)	*N* = 15	Cannabis use inclusion criteria: ND Baseline cannabis use: an average of 16.5 (± 17.1) joints per week	100% male	32 ± 7.1	To determine the effect of marijuana potency on smoking patterns	Sequential design with 2 CSA sessions (same day): placebo cannabis followed by active cannabis 30 min later. Participants were blinded to the potency. Ad libitum procedure: participants were given 1 cannabis cigarette per CSA session. ND on time. Hours of abstinence before session: ≥ 6 h. Abstinence verification: ND.	0.0% and 1.24%	0-100 intoxication rating collected immediately after completion of CSA	“High” rating for 1.24% THC cannabis (mean rating = 54) was significantly higher than placebo cannabis (mean rating = 15).	N/A	Amount consumed: N/A Smoking topography: no differences were found in puff number, puff volume, puff duration, interpuff interval, inhalation volume, and breath-holding time between placebo and active cannabis. Puff volume was significantly lower when participants were smoking the second half of the cigarettes for both the 1.24% THC cigarette and placebo cannabis. No significant difference was found in puff number, puff duration, interpuff interval, inhalation volume, and breath-holding time between the first and second half of the cigarette.	N/A
Zacny and De Wit (1991)	*N* = 5	Cannabis use inclusion criteria: does not meet criteria for substance use disorder (DSM-III) except tobacco dependence Baseline cannabis use: 1-3 times per week	80% male	24 ± ND	“The effects of fasting on the intake and subjective effects of marijuana were studied in five marijuana smokers.”	Cross-over design with 6 CSA sessions (separated by at least 2 or 7 days) using placebo cannabis and two cannabis potencies. Each cannabis potency was administered in a fasting condition (24 h prior to experimental session) and fed condition. Order of CSA sessions was randomized. Participants and staff were blinded to the potency. Prior to the ad libitum period, participants administered “two puffs from each of two half-length marijuana cigarettes (placebo, 0.8%, or 3.6% THC) according to a uniform puffing procedure” directed by a technician. Sixty minutes following the uniformed puffing procedure, the 30-min ad libitum self-administration period started. Ad libitum procedure: participants were given 30 min to smoke up to 10 half-length cannabis cigarettes (1 participant was only given 8 half-length cigarettes). Hours of abstinence before session: ND. Abstinence verification: ND.	0.0%, 0.8%, and 3.6%	6-item VAS and ARCI collected before smoking and 5, 20, and 60 min after uniformed puffing procedure One hour after the uniform puffing procedure, participants were asked to rate peak marijuana effects, peak marijuana high, and marijuana liking.	Ratings of “high” increased with potency level. Peak marijuana effects, peak marijuana high, and marijuana liking also increased with potency level.	20 min post uniformed puffing procedure (high)	Amount consumed: N/A Smoking topography: No difference in puff numbers between the different cannabis potencies or in the different feeding condition.	Hunger, plasma glucose levels, presence of ketone bodies in urine samples, heart rate, expired carbon monoxide levels

*ND* no data, *THC* tetrahydrocannabinol, *11-OH-THC* 11-hydroxy-THC, *THCCOOH* 11-nor-9-carboxy-THC, *VAS* Visual Analog Scale, *POMS* Profile of Mood States, *ARCI* Addictions Research Center Inventory, *DEQ* Drug Effects Questionnaire, *DSM* Diagnostic and Statistical Manual of Mental Disorders

*Study was not fully ad libitum in that subjects were told to smoke a fixed amount of cannabis. However, given that subjects were allowed to smoke the cigarette in any way they wanted and that data derived from the study looked at smoking topography or subjective response, the study was included in our review

**Obtained from author

^CC^Calculated

^a^Total sample size was 91 participants but only 61 participants were randomized to active cannabis (12.5% THC) and the other 30 participants were randomized to placebo cannabis (0.009% THC). One of the 61 participants was excluded from final analysis due to missing subjective data

^b^After the cannabis cigarettes were reassayed, the THC concentration changed to 0.5%, 1.7%, and 2.1%

^c^Data on 5.30% THC was not reported in the manuscript

^d^Total sample size is 191 participants but only 128 participants were randomized to receive active cannabis (66 in 5.90% THC group, 62 in 13.4% THC group), and the other 63 participants were randomized to placebo cannabis

^e^Study’s inclusion criteria, objectives, and study design were obtained from Vandrey et al. (2011)

^f^Study’s male proportion data was obtained from Mirin et al. (1971)

^g^Fourteen participants completed the CSA sessions and only 10 participants completed the brain imaging session. The mean age and standard deviation were based on the 10 participants who completed neuroimaging

## Results

### Study selection

Thirty ad libitum CSA articles were found to be eligible (Fig. 1). Three studies had multiple articles (1 study had 3 papers (Brands et al. 2019; Matheson et al. 2020a, 2020b), and 2 studies had 2 papers each (Hoffman et al. 2021; Marcotte et al. 2022; Spindle et al. 2018, 2019)) resulting in a total of 26 ad libitum studies. Certain included studies were not fully ad libitum in that subjects were told to smoke a fixed amount (Herning et al. 1986; Heishman et al. 1989; Meyer et al. 1971; Miller et al. 1977a, 1977b, 1977c, 1977d, 1978, 1979; Miller and Cornett 1978; Perez-Reyes et al. 1981, 1982; Spindle et al. 2018, 2019, 2021; Schaefer et al. 1977). However, given that subjects were allowed to smoke the cannabis in any way that they wanted and that data derived from the studies looked at puff characteristics, other topography measures, and subjective response, it was felt that these articles contributed valuable information to our review, so the articles were included.

### Study design

The included ad libitum studies were performed in either inpatient or outpatient units with various study designs (i.e., cross-over, sequential, single session, residential, and between-subject designs) and typically consisted of 5-20 participants. One group of investigators conducted studies with slightly larger sample sizes of 28-40 (Miller, Cornett, Brightwell, McFarland, Drew, et al., 1977; Miller, Cornett, Drew, McFarland, Brightwell, et al., 1977; Miller, Cornett, Brightwell, McFarland, Drew, et al., 1977; Miller, Cornett, Drew, McFarland, Brightwell, et al., 1977); the authors had noted that some of the participants were involved in more than one study (Miller & Cornett, 1978). Two studies had much larger sample sizes; one included 91 participants (Brands et al., 2019; Matheson, Mann, et al., 2020; Matheson, Sproule, et al., 2020) and another included 191 participants (Hoffman et al., 2021; Marcotte et al., 2022). In the cross-over and sequential studies, thirteen reported a separation period, usually ranging between 2 days to at least a week (Cappell et al., 1973; Chait, 1989; Heishman et al., 1989; Matthias et al., 1997; Meyer et al., 1971; Miller et al., 1978, 1979; Miller, McFarland, Cornett, Brightwell, et al., 1977; Miller & Cornett, 1978; Perez-Reyes et al., 1982; Spindle et al., 2018, 2019; Zacny & De Wit, 1991). Only one study had a separation period of 24 h (Miller, Cornett, Brightwell, McFarland, Drew, et al., 1977). To prevent residual (carryover) effects from any previous cannabis consumption, participants would often be asked to abstain from cannabis use anywhere from 6 h to 4 days before the ad libitum procedures (Brands et al., 2019; Cappell et al., 1973; Chait, 1989; Heishman et al., 1989; Hoffman et al., 2021; Marcotte et al., 2022; Matheson, Mann, et al., 2020; Matheson, Sproule, et al., 2020; Matthias et al., 1997; Miller et al., 1978; Miller, McFarland, Cornett, Brightwell, et al., 1977; Miller & Cornett, 1978; Wu et al., 1988), although this was not reported in all studies. The longest abstinence period was 11 days of detoxification prior to the study sessions (Tashkin et al., 1976). Abstinence was confirmed by clinical assessments (Herrmann et al., 2015), urine drug screen test (Brands et al., 2019; Matheson, Mann, et al., 2020; Matheson, Sproule, et al., 2020; Spindle et al., 2018, 2019), or oral fluid THC testing (Hoffman et al., 2021; Marcotte et al., 2022). Although most studies (17/26) provided participants with one cannabis cigarette for each ad libitum session, other studies offered multiple cigarettes per session, with cigarettes either provided at the start of the session or requested one at a time from the study staff. One paper had a residential study design with participants having the option to smoke as many cannabis cigarettes as desired for a total of 80 days (Tashkin et al., 1976). The length of the cigarette was not equivalent in all studies and only some studies measured the weight of each cigarette before and after use. Different studies used different instructions, which may also have affected participant behavior (Supplementary Table 3).

### Participants

The study population typically consisted of heavy cannabis users between the ages of 18 and 55 (Table 1). The definition for heavy cannabis user varied across studies, with investigators defining heavy or frequent consumers as using cannabis at least twice per month (Schwope et al., 2012), at least two times per week (Herrmann et al., 2015), four or more times per week (Hoffman et al., 2021; Marcotte et al., 2022), or using cannabis almost daily (e.g., ≥ 25 days/month) (McClure et al., 2012; Meyer et al., 1971; Spindle et al., 2021). Reported baseline cannabis use often significantly exceeded minimum requirements as per inclusion criteria. Although the majority of our included studies did not address whether the participants were treatment-seeking, four studies (Brands et al., 2019; Chait, 1989; Hoffman et al., 2021; Marcotte et al., 2022; Matheson, Mann, et al., 2020; Matheson, Sproule, et al., 2020; Zacny & De Wit, 1991) did indicate that individuals with cannabis dependence or other substance use disorders would be excluded from participating in the study. Most studies (19/26) recruited entirely or predominantly (≥ 80%) male participants.

### Subjective effects

Among the various types of subjective responses, drug “high” or “intoxication” were the most commonly measured. Subjective responses were often measured using a visual analog scale (VAS) or a simple 0-100 scale rating. Peak “high” usually occurred 5-30 min after exposure (Brands et al., 2019; Chait, 1989; Heishman et al., 1989; Herning et al., 1986; Matheson, Mann, et al., 2020; Matheson, Sproule, et al., 2020; Schwope et al., 2012; Spindle et al., 2018, 2019, 2021; Zacny & De Wit, 1991). The time for subjective effects to return to baseline differed across studies, from 4 h (Herrmann et al., 2015) to 6 h or more (Perez-Reyes et al., 1982; Schwope et al., 2012; Spindle et al., 2018, 2019).

### Cannabis consumption

There are various methods for measuring the amount of cannabis consumed, such as the number of cigarettes administered, change in weight of cannabis cigarettes from pre-administration to post-administration, plasma THC levels, and smoking topography variables. Smoking topography examines how cannabis was smoked, such as smoking duration, puff number, and puff volume, which can be captured by observation or using a measurement tool like a single flow transducer, pneumotachograph, or spirometer. Twenty of our included studies assessed for smoking topography while 9 studies reported amount administered, and only 6 studies assessed for both (Brands et al., 2019; Cappell et al., 1973; Herrmann et al., 2015; Hoffman et al., 2021; Marcotte et al., 2022; Matheson, Mann, et al., 2020; Matheson, Sproule, et al., 2020; McClure et al., 2012; Perez-Reyes et al., 1982). In one study, when the participants were given higher potency cannabis (3.90% THC) (Herning et al., 1986), more puffs and larger inhalation volume were taken compared to lower potency cannabis (1.20% THC). In contrast, another study found that when participants smoked a higher potency (2.7% THC), the puff duration and volume were significantly reduced compared to the lower potency cannabis (1.3% THC) (Heishman et al., 1989). Six studies found no significant difference in smoking topography variables between different active THC potencies and between active and placebo cannabis (Cappell et al., 1973; Hoffman et al., 2021; Matthias et al., 1997; Perez-Reyes et al., 1982; Wu et al., 1988; Zacny & De Wit, 1991).

Studies consistently demonstrated that the longer one smokes, the lower the puff volume (Heishman et al., 1989; Herrmann et al., 2015; McClure et al., 2012; Wu et al., 1988). The start of the self-administration period may be the most intense, with puff volume and duration being higher in the first four puffs compared to the last four puffs of the cigarette (McClure et al., 2012). In another study, puff volume decreased 34% in the second half of the hour as compared to the first half (Herrmann et al., 2015). Similarly, when cannabis users were given 9 h of cannabis access, the puff volume and duration decreased with progressive puffs (McClure et al., 2012).

Cannabis consumption was usually measured by the number of cigarettes consumed or the weight of cigarettes before and after smoking. Only two studies reported data on cannabis craving (Herrmann et al., 2015; McClure et al., 2012), although one of these studies (McClure et al., 2012) only looked at craving during a forced abstinence period following cannabis self-administration rather than during the ad libitum session itself.

In terms of smoking duration, CSA sessions were at least 10 min in length. The longest single smoking session was 60 min (Herrmann et al., 2015). The longest ad libitum CSA period was a 94-day study on a closed research unit, where participants underwent 11 days of forced abstinence prior to 80 days of ad libitum use (except between day 76 and 82). In this study, participants consumed an average of 5.2 cannabis cigarettes daily (Tashkin et al., 1976).

Multiple biological specimens can be used as objective indicators of cannabis use such as urine, blood, hair, and oral fluids. However, in human laboratory studies that require multiple samples at different time points, blood samples were most often used given the short detection window that could identify both the parent drug and their metabolites within minutes after exposure (Hadland & Levy, 2016). THC could be detected in the plasma within a minute after the first puff of a cannabis cigarette and peaked within 10 min (Musshoff & Madea, 2006). In our review, only seven studies measured blood THC levels (Brands et al., 2019; Herrmann et al., 2015; Hoffman et al., 2021; Marcotte et al., 2022; Matheson, Mann, et al., 2020; Matheson, Sproule, et al., 2020; Perez-Reyes et al., 1981, 1982; Schwope et al., 2012; Spindle et al., 2018, 2019). Three of these studies reported that peak subjective response (e.g., “high,” “stimulated,” “stoned,” “good drug effects,” “drug effects”) occurred when plasma THC levels peaked (Matheson, Sproule, et al., 2020; Schwope et al., 2012; Spindle et al., 2018, 2019). However, two studies with smaller sample size found that peak subjective response occurred after peak plasma THC levels were reached (Perez-Reyes et al., 1981, 1982).

### Cannabis withdrawal

Cannabis withdrawal may begin within 1 day of abstinence (Budney et al., 2007; Connor et al., 2022), so some cannabis users may have already been in withdrawal at the start of the paradigm given that most ad libitum CSA studies required the participants to abstain from cannabis use before entering the laboratory. However, most studies did not measure withdrawal symptoms prior to self-administration to see if this impacted craving, subjective response, or self-administration behavior. Although one study (Spindle et al., 2021) did administer the Marijuana Withdrawal Checklist prior to the CSA session, the authors did not report whether it had any effect on subjective response or CSA behavior.

### Test-retest reliability and external validity

There was limited data on the test-retest reliability and external validity of laboratory CSA. Although two studies (Chait, 1989; McClure et al., 2012) in our review had participants repeat the CSA sessions under the same conditions, neither study reported correlations in CSA behavior between the test sessions. One study (McClure et al., 2012) found that frequency of daily cannabis use in the past 30 days prior to the study was positively associated with total puff volume and maximum puff duration but no other smoking topography measures. Furthermore, the study found a correlation between years of cannabis use and total puff volume per cigarette, average volume per puff, and average puff duration. In a driving stimulation study (Hoffman et al., 2021; Marcotte et al., 2022), cannabis users with higher intensity of cannabis use in the past 6 months had the highest whole blood THC concentration post-smoking. This finding suggests that whole blood THC concentration during ad libitum consumption may be correlated with frequency of cannabis use outside of the laboratory.

### Effects of cannabis potency

Compared to earlier studies, the potency of cannabis used in recent CSA studies has increased, with potencies as high as 13.4% being used (Hoffman et al., 2021; Marcotte et al., 2022; Spindle et al., 2018, 2019, 2021). Although higher potency cannabis resulted in significantly higher subjective responses (particularly “high” ratings) than their lower potency counterparts in most of our included studies (Cappell et al., 1973; Herning et al., 1986; Miller, Cornett, Drew, McFarland, Brightwell, et al., 1977; Miller & Cornett, 1978; Perez-Reyes et al., 1982; Schaefer et al., 1977; Zacny & De Wit, 1991), the largest study included in our review (*N* = 191) (Hoffman et al., 2021; Marcotte et al., 2022) found that individuals reported greater “high” in the 5.9% THC group than in the 13.4% THC group. The 5.9% THC group also achieved the highest blood THC concentration among the three potency groups (placebo, 5.9% THC, and 13.4% THC). Three papers (Chait, 1989; Heishman et al., 1989; Matthias et al., 1997) reported no significant difference in subjective response between the various cannabis potencies; however, this may have been due to the narrow concentration range (2.7% vs. 1.7% vs. 0.9% THC (Chait, 1989); 3.95% vs. 1.77% vs. 0.0% THC (Matthias et al., 1997); 2.7% vs. 1.3% (Heishman et al., 1989), making it difficult for participants to differentiate the various cannabis concentrations. These studies also had small sample sizes and were potentially underpowered to detect differences in subjective response between potencies.

Even though subjective response increases in a dose-dependent manner in some studies, there may still be no difference in smoking topography variables (Cappell et al., 1973; Matthias et al., 1997; Perez-Reyes et al., 1982; Zacny & De Wit, 1991) and amount consumed (Chait, 1989; Hoffman et al., 2021; Marcotte et al., 2022; Perez-Reyes et al., 1982). Only two studies in our review showed that cannabis potency affected smoking topography variables (e.g., puff and inhalation volume) (Heishman et al., 1989; Herning et al., 1986) and one study found an inverse relationship between cannabis potencies and amount smoked (Cappell et al., 1973). More research is required to determine cannabis potency’s effect on smoking topography variables and cannabis consumption.

### Effects of sex

Included studies had a higher proportion of male participants. Only four studies examined sex differences between cannabis users (Matheson, Sproule, et al., 2020; Perez-Reyes et al., 1981, 1982; Spindle et al., 2019). Despite smoking for the same duration as male participants, female participants were found to smoke less of the cigarette, suggesting the female participants may be taking smaller and less frequent puffs than the male participants (Matheson, Sproule, et al., 2020). This supports an earlier small study (3 males, 3 females) which found that males took more puffs and consumed cigarettes more quickly than females, although it was not indicated whether these differences were statistically significant (Perez-Reyes et al., 1981). However, a later study found no difference in smoking pattern between the two sexes (Perez-Reyes et al., 1982). It has also been reported that females attain significantly lower maximum blood THC concentration than males (Matheson, Sproule, et al., 2020) and that females had numerically lower area under the plasma THC versus time curve than males (although the difference was not statistically significant) (Perez-Reyes et al., 1981). In contrast, two studies found that females achieved numerically higher blood THC concentrations than males (Perez-Reyes et al., 1982; Spindle et al., 2019). Although few differences in subjective response were identified between the sexes, “liking” and “feels like cannabis” ratings (Matheson, Sproule, et al., 2020) were higher in males. Despite having the same peak time and ratings, females’ ratings rapidly declined at 180 min and returned to baseline at 360 min (Matheson, Sproule, et al., 2020). Meanwhile, subjective response in males tended to persist longer, as they still had significantly different “liking” and “feels like cannabis” ratings from baseline at 360 min (Matheson, Sproule, et al., 2020).

### Effects of environment

Few studies have examined the impact of surroundings and environment on CSA behavior. Some examples of potential influences could include the size and furnishings of the smoking room and the presence of other people (e.g., study staff or other cannabis smokers) in the room during self-administration. The influence of room ventilation on physiological, subjective, and behavioral effects of cannabis smoking was examined in one study (Herrmann et al., 2015). A group of cannabis smokers consumed a total of 2.1 g more cannabis in the ventilated condition (16.5 g total) than the unventilated condition (14.4 g total), but the authors did not address whether this was a statistically significant difference. There appeared to be no difference in subjective response (“feel drug effect”) by ventilation condition (Herrmann et al., 2015). Some studies had participants smoke in groups (Herrmann et al., 2015; Miller, Cornett, Brightwell, McFarland, Drew, et al., 1977; Miller, Cornett, Drew, McFarland, Brightwell, et al., 1977; Miller et al., 1979; Miller, McFarland, Cornett, & Brightwell, 1977), but it is unknown if this may have impacted smoking behavior.

## Discussion

The objective of our scoping review was to provide an overview of ad libitum CSA studies as well as the limitations of these studies. From our included studies, we found there was a high level of heterogeneity in ad libitum study designs, with differences in self-administration instructions, length of administration, and smoking environment. These factors could influence CSA outcomes; however, it is difficult to determine how important these factors are given the lack of comparative research. Outcome variables also differed between studies. For example, some ad libitum studies measure smoking topography variables such as puff number, puff volume, and inhalation duration, while others only measure the number of cigarettes smoked or the weight of the cigarette before and after smoking. The differences in design and measurement make it challenging to compare across studies.

The lack of major differences in smoking topography outcomes between different cannabis potencies (Hoffman et al., 2021; Matthias et al., 1997; Wu et al., 1988; Zacny & De Wit, 1991) is surprising. The few studies that found differences between cannabis potencies (Heishman et al., 1989; Herning et al., 1986) suggest that some smoking titration may be involved. The study with the largest sample size reported that individuals experienced a greater “high” in the 5.9% THC group compared to the 13.4% THC group (Hoffman et al., 2021; Marcotte et al., 2022), suggesting that potency may not be the only factor that determines “high,” but other factors (e.g., inhalation volume) might play an important role (especially since individuals in the 5.9% THC group had higher blood THC concentrations than those in the 13.4% THC group in this study).

None of the papers we reviewed examined the effects of age on subjective response, cannabis consumption, or smoking topography. Most study samples consisted of adults less than 40 years of age, with few studies examining CSA in older adults. The use of cannabis in adults age 65 and older has been gradually increasing from 0.4% in 2006 (Han et al., 2017) to about 2.9% by 2016 (Han & Palamar, 2018). A recent study (Mueller et al., 2021) with recreational users reported that THC-dominant cannabis use in older adults (age 55-70) had less detrimental effects on learning and processing speed tests than in younger adults (age 21-25). However, older adults may be more likely to develop sedation, reduced consciousness, lightheadedness, and weakness/inability to stand after acute cannabis exposure compared to younger adults (age 19-59) (Hendrickson et al., 2020), which may require more attention and care during CSA. Cannabis use has also been linked to higher odds of myocardial infarction, coronary artery disease, and stroke in older adults (Shah et al., 2021) who are already more vulnerable to cardiovascular events (Latif & Garg, 2020; Rodgers et al., 2019). This may be a barrier to studying cannabis self-administration in older adults, especially in individuals at heightened risk of potential cardiovascular sequelae, such as those with pre-existing cardiovascular disease.

Subjects in ad libitum paradigms were also predominately male, with the percentage of male participants usually ranging between 70 and 100%. Given varying CSA behaviors in females and males in preclinical research (Fattore et al., 2007), it is important that females be represented in this literature. Only four ad libitum studies examined the effects of sex on subjective response and cannabis consumption (Matheson, Sproule, et al., 2020; Perez-Reyes et al., 1981, 1982; Spindle et al., 2019). In these studies, findings on smoking behavior, subjective response, and pharmacokinetic profiles between the sexes were contradictory (Matheson, Sproule, et al., 2020; Perez-Reyes et al., 1981, 1982; Spindle et al., 2019). Clearly, more research comparing CSA behavior in males and females is needed.

Other factors that may impact CSA outcomes are withdrawal, acute craving, tolerance, sleep quality, stress, anxiety, and mood. These factors have been understudied in ad libitum CSA paradigms to date. One study found a negative correlation between puff duration and sleep quality (McClure et al., 2012). Only one study assessed cannabis withdrawal, but this study did not report whether there were any associations with subjective response or consumption (Spindle et al., 2021).

Perhaps most importantly, our review reveals limited assessment of test-retest reliability and external validity for most ad libitum CSA paradigms. From our included ad libitum studies, only two studies repeated CSA paradigms at least twice under the same condition on different days (Chait, 1989; McClure et al., 2012); however, neither study reported whether consumption behavior or subjective response were correlated between sessions. Only two studies (Hoffman et al., 2021; Marcotte et al., 2022; McClure et al., 2012) examined the validity of laboratory cannabis consumption by comparing the puff volumes or blood THC measures during the ad libitum period with external consumption. Insufficient verification of external validity raises the possibility that laboratory results might be an inaccurate representation of real-world CSA behavior; therefore, more data on the reliability and validity of these paradigms is critical to determine their real-world usefulness. Using validated self-report assessments (e.g., Timeline Followback, Marijuana Craving Questionnaire) or real-time report (e.g., Ecological Momentary Assessment (Trull et al., 2022)) prior to the CSA session and comparing these measures to related outcomes in the laboratory could help confirm external validity. Test-retest reliability could be measured using within-subject designs where participants repeat the same CSA session at two or more time points to see if self-administration behavior, peak THC levels achieved, craving, and subjective response are correlated between the sessions and the magnitude of these correlations.

Our review is limited by the fact that it focused primarily on ad libitum paradigms that investigated subjective response and self-administration behavior. Reviews focused on other outcomes such as medication effects or driving are needed to determine whether CSA paradigms are reliable and externally valid for those specific outcomes. Some cannabis-related outcomes might be better assessed by employing other types of drug self-administration paradigms such as controlled-smoking procedures or choice procedures where individuals pay for access to cannabis, but these were not reviewed here.

Based on the results of our review, we found that there is a high level of heterogeneity across ad libitum CSA studies. Self-administration behavior in ad libitum studies appeared to be most intense in the early part of laboratory sessions and decreased in the latter parts of the session, suggesting that users may reach their desired high early on during self-administration. Data on test-retest reliability and external validity were limited. Test-retest reliability and external validity data should be collected when developing and evaluating novel paradigms to ensure that they reliably reflect real-world CSA behavior. CSA studies in older adult and female samples are needed to better understand cannabis administration in these demographic groups. Future ad libitum CSA studies should also collect data on craving and cannabis withdrawal to determine the impact of these measures on self-administration. More thoughtful design of ad libitum CSA studies could lead to better quality data and improved paradigms, which may help us understand why certain individuals are at risk for developing CUD and lead to effective platforms to test novel pharmacotherapies and interventions for cannabis use disorder.

## Supplementary information

ESM 1: Supplementary Table 1.1. PubMed Search Strategy Conducted on October 22, 2022. Supplementary Table 1.2. Embase Search Strategy Conducted on October 22, 2022. Supplementary Table 1.3. Initial PUBMED Search Strategy Conducted on March 7, 2021. Supplementary Table 2. Excluded CSA Ad libitum articles (n= 34). Supplementary Table 3. Instructions Used for Ad Libitum smoking (DOCX 41 kb)

## References

[CR1] Blanco C, Hasin DS, Wall MM, Flórez-Salamanca L, Hoertel N, Wang S, Kerridge BT, Olfson M (2016). Cannabis use and risk of psychiatric disorders: prospective evidence from a US national longitudinal study. JAMA Psychiatr.

[CR2] Brands B, Mann RE, Wickens CM, Sproule B, Stoduto G, Sayer GS, Burston J, Pan JF, Matheson J, Stefan C, George TP, Huestis MA, Rehm J, Le Foll B (2019). Acute and residual effects of smoked cannabis: impact on driving speed and lateral control, heart rate, and self-reported drug effects. Drug Alcohol Depend.

[CR3] Budney AJ, Roffman R, Stephens RS, Walker D (2007). Marijuana dependence and its treatment. Addiction Science & Clinical Practice.

[CR4] Cappell H, Kuchar E, Webster CD (1973). Some correlates of marihuana self-administration in man: a study of titration of intake as a function of drug potency. Psychopharmacologia.

[CR5] Chait LD (1989). Delta-9-tetrahydrocannabinol content and human marijuana self-administration. Psychopharmacology.

[CR6] Chukwueke CC, Le Foll B (2019). The human laboratory and drug development in alcohol use disorder: recent updates. Methods in Molecular Biology (Clifton, NJ).

[CR7] Connor JP, Stjepanović D, Le Foll B, Hoch E, Budney AJ, Hall WD (2021). Cannabis use and cannabis use disorder. Nature Reviews Disease Primers.

[CR8] Connor JP, Stjepanović D, Budney AJ, Le Foll B, Hall WD (2022). Clinical management of cannabis withdrawal. Addiction.

[CR9] European Monitoring Centre for Drugs and Drug Addiction (2021) Cannabis: health and social responses. In Health and social responses to drug problems: A European guide 2021. https://www.emcdda.europa.eu/publications/mini-guides/cannabis-health-and-social-responses_en#section3

[CR10] Fattore L, Spano MS, Altea S, Angius F, Fadda P, Fratta W (2007). Cannabinoid self-administration in rats: sex differences and the influence of ovarian function. Br J Pharmacol.

[CR11] Fogel JS, Kelly TH, Westgate PM, Lile JA (2017). Sex differences in the subjective effects of oral Δ9-THC in cannabis users. Pharmacol Biochem Behav.

[CR12] Gowin JL, Sloan ME, Stangl BL, Vatsalya V, Ramchandani VA (2017). Vulnerability for alcohol use disorder and rate of alcohol consumption. Am J Psychiatr.

[CR13] Grotenhermen F (2003). Pharmacokinetics and pharmacodynamics of cannabinoids. Clin Pharmacokinet.

[CR14] Hadland SE, Levy S (2016). Objective testing: urine and other drug tests. Child Adolesc Psychiatr Clin N Am.

[CR15] Han BH, Palamar JJ (2018). Marijuana use by middle-aged and older adults in the United States, 2015-2016. Drug Alcohol Depend.

[CR16] Han BH, Sherman S, Mauro PM, Martins SS, Rotenberg J, Palamar JJ (2017). Demographic trends among older cannabis users in the United States, 2006-13. Addiction.

[CR17] Haney M (2009). Self-administration of cocaine, cannabis and heroin in the human laboratory: benefits and pitfalls. Addict Biol.

[CR18] Hasin DS, Saha TD, Kerridge BT, Goldstein RB, Chou SP, Zhang H, Jung J, Pickering RP, Ruan WJ, Smith SM, Huang B, Grant BF (2015). Prevalence of marijuana use disorders in the United States between 2001-2002 and 2012-2013. JAMA Psychiatry.

[CR19] Health Canada (2021) Canadian Cannabis Survey 2020: Summary. https://www.canada.ca/en/health-canada/services/drugs-medication/cannabis/research-data/canadian-cannabis-survey-2020-summary.html

[CR20] Heishman SJ, Stitzer ML, Yingling JE (1989). Effects of tetrahydrocannabinol content on marijuana smoking behavior, subjective reports, and performance. Pharmacol Biochem Behav.

[CR21] Herning RI, Hooker WD, Jones RT (1986) Tetrahydrocannabinol content and differences in marijuana smoking behavior. Psychopharmacology 90(2). 10.1007/BF00181232 10.1007/BF00181232 3024196

[CR22] Hendrickson RG, McKeown NJ, Kusin SG, Lopez AM (2020). Acute cannabis toxicity in older adults. Toxicology Communications.

[CR23] Herrmann ES, Cone EJ, Mitchell JM, Bigelow GE, LoDico C, Flegel R, Vandrey R (2015). Non-smoker exposure to secondhand cannabis smoke II: effect of room ventilation on the physiological, subjective, and behavioral/cognitive effects. Drug Alcohol Depend.

[CR24] Hoffman MA, Hubbard JA, Sobolesky PM, Smith BE, Suhandynata RT, Sanford S, Sones EG, Ellis S, Umlauf A, Huestis MA, Grelotti DJ, Grant I, Marcotte TD, Fitzgerald RL (2021). Blood and oral fluid cannabinoid profiles of frequent and occasional cannabis smokers. J Anal Toxicol.

[CR25] Jones JD, Comer SD (2013). A review of human drug self-administration procedures. Behav Pharmacol.

[CR26] Kayser RR, Haney M, Simpson HB (2021). Human laboratory models of cannabis use: applications for clinical and translational psychiatry research. Frontiers in Psychiatry.

[CR27] Kuepper R, van Os J, Lieb R, Wittchen H-U, Hofler M, Henquet C (2011). Continued cannabis use and risk of incidence and persistence of psychotic symptoms: 10 year follow-up cohort study. BMJ.

[CR28] Latif Z, Garg N (2020). The impact of marijuana on the cardiovascular system: a review of the most common cardiovascular events associated with marijuana use. J Clin Med.

[CR29] Marcotte TD, Umlauf A, Grelotti DJ, Sones EG, Sobolesky PM, Smith BE, Hoffman MA, Hubbard JA, Severson J, Huestis MA, Grant I, Fitzgerald RL (2022). Driving performance and cannabis users’ perception of safety: a randomized clinical trial. JAMA Psychiatry.

[CR30] Matheson J, Mann RE, Sproule B, Huestis MA, Wickens CM, Stoduto G, George TP, Rehm J, Le Foll B, Brands B (2020). Acute and residual mood and cognitive performance of young adults following smoked cannabis. Pharmacol Biochem Behav.

[CR31] Matheson J, Sproule B, Di Ciano P, Fares A, Le Foll B, Mann RE, Brands B (2020). Sex differences in the acute effects of smoked cannabis: evidence from a human laboratory study of young adults. Psychopharmacology.

[CR32] Matthias P, Tashkin DP, Marques-Magallanes JA, Wilkins JN, Simmons MS (1997) Effects of varying marijuana potency on deposition of tar and ⌬9-THC in the lung during smoking. 6 10.1016/s0091-3057(97)00328-6 9408226

[CR33] McClure EA, Stitzer ML, Vandrey R (2012). Characterizing smoking topography of cannabis in heavy users. Psychopharmacology.

[CR34] McKee SA (2009). Developing human laboratory models of smoking lapse behavior for medication screening. Addict Biol.

[CR35] Meyer RE, Pillard RC, Shapiro LM, Mirin SM (1971). Administration of marijuana to heavy and casual marijuana users. Am J Psychiatr.

[CR36] Miller LL, Cornett TL (1978). Marijuana: dose effects on pulse rate, subjective estimates of intoxication, free recall and recognition memory. Pharmacol Biochem Behav.

[CR37] Miller LL, Cornett TL, Brightwell DR, McFarland DJ, Drew WD, Wikler A (1977). Marijuana: effects on storage and retrieval of prose material. Psychopharmacology.

[CR38] Miller LL, Cornett TL, Drew WD, McFarland DJ, Brightwell DR, Wikler A (1977). Marijuana: dose-response effects on pulse rate, subjective estimates of potency, pleasantness, and recognition memory. Pharmacology.

[CR39] Miller LL, McFarland DJ, Cornett TL, Brightwell DR (1977). Marijuana and memory impairment: effect on free recall and recognition memory. Pharmacol Biochem Behav.

[CR40] Miller LL, McFarland DJ, Cornett TL, Brightwell DR, Wikler A (1977). Marijuana: effects on free recall and subjective organization of pictures and words. Psychopharmacology.

[CR41] Miller LL, Cornett TL, McFarland DJ (1978). Marijuana: an analysis of storage and retrieval deficits in memory with the technique of restricted reminding. Pharmacol Biochem Behav.

[CR42] Miller LL, Cornett TL, Wikler A (1979). Marijuana: effects on pulse rate, subjective estimates of intoxication and multiple measures of memory. Life Sci.

[CR43] Mirin SM, Shapiro LM, Meyer RE, Pillard RC, Fisher S (1971). Casual versus heavy use of marijuana: a redefinition of the marijuana problem. Am J Psychiatry.

[CR44] Mueller RL, Ellingson JM, Bidwell LC, Bryan AD, Hutchison KE (2021). Are the acute effects of THC different in aging adults?. Brain Sciences.

[CR45] Musshoff F, Madea B (2006). Review of biologic matrices (urine, blood, hair) as indicators of recent or ongoing cannabis use. Ther Drug Monit.

[CR46] NIDA (2021) Is there a link between marijuana use and psychiatric disorders? https://www.drugabuse.gov/publications/research-reports/marijuana/there-link-between-marijuana-use-psychiatric-disorders

[CR47] Pacher P, Bátkai S, Kunos G (2006). The endocannabinoid system as an emerging target of pharmacotherapy. Pharmacol Rev.

[CR48] Panlilio LV, Justinova Z, Trigo JM, Le Foll B (2016). Screening medications for the treatment of cannabis use disorder. Int Rev Neurobiol.

[CR49] Perez-Reyes M, Owens SM, Di Guiseppi S (1981). The clinical pharmacology and dynamics of marihuana cigarette smoking. J Clin Pharmacol.

[CR50] Perez-Reyes M, Guiseppi SD, Davis KH, Schindler VH, Edgar Cook C (1982). Comparison of effects of marihuana cigarettes of three different potencies. Clin Pharmacol Ther.

[CR51] Ray LA, Du H, Green R, Roche DJO, Bujarski S (2021). Do behavioral pharmacology findings predict clinical trial outcomes? A proof-of-concept in medication development for alcohol use disorder. Neuropsychopharmacology: Official Publication of the American College of Neuropsychopharmacology.

[CR52] Rodgers JL, Jones J, Bolleddu SI, Vanthenapalli S, Rodgers LE, Shah K, Karia K, Panguluri SK (2019). Cardiovascular risks associated with gender and aging. Journal of Cardiovascular Development and Disease.

[CR53] Russell C, Rueda S, Room R, Tyndall M, Fischer B (2018). Routes of administration for cannabis use – basic prevalence and related health outcomes: a scoping review and synthesis. Int J Drug Policy.

[CR54] SAMHSA (2021). 2020 National Survey of Drug Use and Health (NSDUH): detailed tables.

[CR55] Schaefer CF, Gunn CG, Dubowski KM (1977). Dose-related heart-rate, perceptual, and decisional changes in man following marihuana smoking. Percept Mot Skills.

[CR56] Schwope DM, Bosker WM, Ramaekers JG, Gorelick DA, Huestis MA (2012). Psychomotor performance, subjective and physiological effects and whole blood 9-tetrahydrocannabinol concentrations in heavy, chronic cannabis smokers following acute smoked cannabis. J Anal Toxicol.

[CR57] Shah S, Patel S, Paulraj S, Chaudhuri D (2021). Association of marijuana use and cardiovascular disease: a behavioral risk factor surveillance system data analysis of 133,706 US adults. Am J Med.

[CR58] Sloan ME, Grant CW, Gowin JL, Ramchandani VA, Le Foll B (2019). Endocannabinoid signaling in psychiatric disorders: a review of positron emission tomography studies. Acta Pharmacol Sin.

[CR59] Sloan ME, Gowin JL, Janakiraman R, Ester CD, Stoddard J, Stangl B, Ramchandani VA (2020). High-risk social drinkers and heavy drinkers display similar rates of alcohol consumption. Addict Biol.

[CR60] Sloan ME, Sells JR, Vaughan CL, Morris JK, Ortega NE, Sundar S, Soundararajan S, Stangl BL, Gowin J, Chawla S, Diazgranados N, McKee SA, Waters A, Ramchandani VA (2022). Modeling ability to resist alcohol in the human laboratory: a pilot study. Drug and Alcohol Dependence Reports.

[CR61] Sorkhou M, Bedder RH, George TP (2021). The behavioral sequelae of cannabis use in healthy people: a systematic review. Frontiers in Psychiatry.

[CR62] Spindle TR, Cone EJ, Schlienz NJ, Mitchell JM, Bigelow GE, Flegel R, Hayes E, Vandrey R (2018). Acute effects of smoked and vaporized cannabis in healthy adults who infrequently use cannabis: a crossover trial. JAMA Netw Open.

[CR63] Spindle TR, Cone EJ, Schlienz NJ, Mitchell JM, Bigelow GE, Flegel R, Hayes E, Vandrey R (2019). Acute pharmacokinetic profile of smoked and vaporized cannabis in human blood and oral fluid. J Anal Toxicol.

[CR64] Spindle TR, Kuwabara H, Eversole A, Nandi A, Vandrey R, Antoine DG, Umbricht A, Guarda AS, Wong DF, Weerts EM (2021) Brain imaging of cannabinoid type I (CB _1_ ) receptors in women with cannabis use disorder and male and female healthy controls. Addict Biol 26(6). 10.1111/adb.13061 10.1111/adb.13061 PMC8516687 34028926

[CR65] Stangl BL, Byrd ND, Soundararajan S, Plawecki MH, O’Connor S, Ramchandani VA (2022) The motivation for alcohol reward: predictors of progressive-ratio intravenous alcohol self-administration in humans. J Visualized Experiments: JoVE 182. 10.3791/63576 10.3791/63576 35575525

[CR66] Tashkin DP, Shapiro BJ, Lee YE, Harper CE (1976). Subacute effects of heavy marihuana smoking on pulmonary function in healthy men. N Engl J Med.

[CR67] Trull TJ, Freeman LK, Fleming MN, Vebares TJ, Wycoff AM (2022). Using ecological momentary assessment and a portable device to quantify standard tetrahydrocannabinol units for cannabis flower smoking. Addiction.

[CR68] Vandrey R, Smith MT, McCann UD, Budney AJ, Curran EM (2011). Sleep disturbance and the effects of extended-release zolpidem during cannabis withdrawal. Drug Alcohol Depend.

[CR69] Veritas Health Innovation (2021) Covidence better systematic review management. https://www.covidence.org

[CR70] Vinette B, Côté J, El-Akhras A, Mrad H, Chicoine G, Bilodeau K (2022). Routes of administration, reasons for use, and approved indications of medical cannabis in oncology: a scoping review. BMC Cancer.

[CR71] Volkow ND, Baler RD, Compton WM, Weiss SRB (2014). Adverse health effects of marijuana use. N Engl J Med.

[CR72] Wu T-C, Tashkin DP, Rose JE, Djahed B (1988). Influence of marijuana potency and amount of cigarette consumed on marijuana smoking pattern. J Psychoactive Drugs.

[CR73] Zacny JP, De Wit H (1991). Effects of food deprivation on subjective effects and self-administration of marijuana in humans. Psychol Rep.

[CR74] Zou S, Kumar U (2018). Cannabinoid receptors and the endocannabinoid system: signaling and function in the central nervous system. Int J Mol Sci.

