# Supplementary material for: Cannabis self-administration in the human laboratory: a scoping review of ad libitum studies
Source: Psychopharmacology (Berl). 2023 May 9;240(7):1393–415. doi: 10.1007/s00213-023-06360-4 (PMC10272254; doi:10.1007/s00213-023-06360-4)
Supplement: Supplementary file 1 — Supplementary Table 1.1. PubMed Search Strategy Conducted on October 22, 2022. Supplementary Table 1.2. Embase Search Strategy Conducted on October 22, 2022. Supplementary Table 1.3. Initial PUBMED Search Strategy Conducted on March 7, 2021. Supplementary Table 2. Excluded CSA Ad libitum articles (n= 34). Supplementary Table 3. Instructions Used for Ad Libitum smoking (DOCX 41 kb) [file 213_2023_6360_MOESM1_ESM.docx]

**Supplementary Table 1.1. PubMed Search Strategy Conducted on October 22, 2022**

| Search # | Query | Items found |
| --- | --- | --- |
| 1 | (“Marijuana Smoking"[Mesh] ) OR ( "Marijuana Abuse"[Mesh]) OR “cannabis users” OR "cannabis smokers" OR "marijuana users" OR "marijuana smokers" OR “smoking blunt*” OR “cannabis joint*” | 13,053 |
| 2 | “cannabis”[Mesh] OR (cannabis OR marijuana OR marihuana) | 42,121 |
| 3 | “Self administration" OR “self use” OR "ad libitum"[tiab] OR "ad lib"[tiab] OR freely[tiab] OR natural*[tiab] OR “free access”[tiab] OR "self-administ*"[tiab] | 923,082 |
| **4** | **(#1 OR #2) AND #3** | **1,820** |

**Supplementary Table 1.2. Embase Search Strategy Conducted on October 22, 2022**

| Search # | Query | Items found |
| --- | --- | --- |
| 1 | ((marijuana or cannabis) and (smok* or users)).mp. | 22,868 |
| 2 | cannabis smoking.mp or exp cannabis smoking/ or exp cannabis/ or exp "cannabis use"/ | 51,871 |
| 3 | self administration.mp. or exp drug self administration/ | 20,452 |
| 4 | (ad libitum or ad lib or freely or natural* or free access) | 1,143,869 |
| 5 | 1 OR 2 | 55,598 |
| 6 | 3 OR 4 | 1,163,275 |
| **7** | **5 AND 6** | **1,888** |

*Note: additional searches were run looking for results related to reliability or validity. On PubMed, we used the following search terms "Reproducibility of Results"[Mesh] OR Reliabilit*[tiab] OR Validit*[tiab] OR “Test-Retest”[tiab] OR “reproduc*”[tiab]. On Embase, we used the following search terms: exp reproducibility/ OR test retest reliability/ OR exp external validity/ OR exp validity/.

**Supplementary Table 1.3. Initial PUBMED Search Strategy Conducted on March 7, 2021**

| Search # | Query | Items found |
| --- | --- | --- |
| 1 | (“Marijuana Smoking"[Mesh] ) OR ( "Marijuana Abuse/drug therapy"[Mesh] OR "Marijuana Abuse/psychology"[Mesh] OR "Marijuana Abuse/rehabilitation"[Mesh] ) | 7,165 |
| 2 | “cannabis”[Mesh] OR (cannabis OR marijuana OR marihuana) AND ("self-administ*" OR smok*) | 10,254 |
| 3 | "smoking pattern"[tiab] OR "smoking behavior"[tiab] OR "ad libitum"[tiab] OR "ad lib"[tiab] OR freely[tiab] OR natural*[tiab] | 768,472 |
| 4 | "Self administration" [Mesh] OR relapse[tiab] OR choice[tiab] OR reinforc*[tiab] | 542,849 |
| 5 | (#1 OR #2) AND #3 | 311 |
| 6 | (#1 OR #2) AND #4 | 560 |
| **7** | **#5 OR #6** | **829** |

**Supplementary Table 2. Excluded CSA Ad libitum articles (n= 34)**

| **Reason for Exclusion** | **Study Title** |
| --- | --- |
| Self-supplied Cannabis (n = 3) | 1. Bidwell, L. C., Ellingson, J. M., Karoly, H. C., YorkWilliams, S. L., Hitchcock, L. N., Tracy, B. L., Klawitter, J., Sempio, C., Bryan, A. D., & Hutchison, K. E. (2020). Association of Naturalistic Administration of Cannabis Flower and Concentrates With Intoxication and Impairment. *JAMA Psychiatry, 77*(8), 787. https://doi.org/10.1001/jamapsychiatry.2020.0927 2. DeGregorio, M. W., Wurz, G. T., Montoya, E., & Kao, C.-J. (2021). A comprehensive breath test that confirms recent use of inhaled cannabis within the impairment window*. Scientific Reports, 11*(1), 22776. <https://doi.org/10.1038/s41598-021-02137-x> 3. Wurz, G. T., & DeGregorio, M. W. (2022). Indeterminacy of cannabis impairment and ∆9-tetrahydrocannabinol (∆9-THC) levels in blood and breath*. Scientific Reports, 12*(1), 8323. https://doi.org/10.1038/s41598-022-11481-5 |
| No CSA behavior or subjective responses (n = 14) | 1. Desrosiers, N. A., Himes, S. K., Scheidweiler, K. B., Concheiro-Guisan, M., Gorelick, D. A., & Huestis, M. A. (2014). Phase I and II cannabinoid disposition in blood and plasma of occasional and frequent smokers following controlled smoked cannabis. *Clinical Chemistry, 60*(4), 631–643. https://doi.org/10.1373/clinchem.2013.216507 2. Desrosiers, N. A., Lee, D., Concheiro-Guisan, M., Scheidweiler, K. B., Gorelick, D. A., & Huestis, M. A. (2014). Urinary cannabinoid disposition in occasional and frequent smokers: Is THC-glucuronide in sequential urine samples a marker of recent use in frequent smokers? *Clinical Chemistry, 60*(2), 361–372. https://doi.org/10.1373/clinchem.2013.214106 3. Desrosiers, N. A., Lee, D., Scheidweiler, K. B., Concheiro-Guisan, M., Gorelick, D. A., & Huestis, M. A. (2014). In Vitro Stability of Free and Glucuronidated Cannabinoids in Urine Following Controlled Smoked Cannabis. *Analytical and Bioanalytical Chemistry, 406*(3), 785–792. https://doi.org/10.1007/s00216-013-7524-7 4. Desrosiers, N. A., Lee, D., Schwope, D. M., Milman, G., Barnes, A. J., Gorelick, D. A., & Huestis, M. A. (2012). On-site test for cannabinoids in oral fluid*. Clinical Chemistry, 58(*10), 1418–1425. https://doi.org/10.1373/clinchem.2012.189001 5. Desrosiers, N. A., Milman, G., Mendu, D. R., Lee, D., Barnes, A. J., Gorelick, D. A., & Huestis, M. A. (2014). Cannabinoids in oral fluid by on-site immunoassay and by GC-MS using two different oral fluid collection devices. *Analytical and Bioanalytical Chemistry, 406*(17), 4117–4128. https://doi.org/10.1007/s00216-014-7813-9 6. Hubbard, J. A., Hoffman, M. A., Ellis, S. E., Sobolesky, P. M., Smith, B. E., Suhandynata, R. T., Sones, E. G., Sanford, S. K., Umlauf, A., Huestis, M. A., Grelotti, D. J., Grant, I., Marcotte, T. D., & Fitzgerald, R. L. (2021). Biomarkers of Recent Cannabis Use in Blood, Oral Fluid and Breath. *Journal of Analytical Toxicology, 45(*8), 820–828. https://doi.org/10.1093/jat/bkab080 7. Lee, D., Schwope, D. M., Milman, G., Barnes, A. J., Gorelick, D. A., & Huestis, M. A. (2012). Cannabinoid disposition in oral fluid after controlled smoked cannabis. *Clinical Chemistry, 58*(4), 748–756. <https://doi.org/10.1373/clinchem.2011.177881> 8. Milman, G., Schwope, D. M., Gorelick, D. A., & Huestis, M. A. (2012). Cannabinoids and Metabolites in Expectorated Oral Fluid Following Controlled Smoked Cannabis. *Clinica Chimica Acta; International Journal of Clinical Chemistry, 413*(7–8), 765–770. <https://doi.org/10.1016/j.cca.2012.01.011> 9. Mosaed, S., Liu, J. H., Minckler, D. S., Fitzgerald, R. L., Grelotti, D., Sones, E., Shiels, C. R., Weinreb, R. N., & Marcotte, T. D. (2021). The Effect of Inhaled Cannabis on Intraocular Pressure in Healthy Adult Subjects. *Ophthalmology, 15*(1), 33. https://doi.org/10.17925/OPHT.2021.15.1.33 10. Mosaed, S., Smith, A. K., Liu, J. H. K., Minckler, D. S., Fitzgerald, R. L., Grelotti, D., Sones, E., Weinreb, R. N., & Marcotte, T. D. (2022). The Relationship Between Plasma Tetrahydrocannabinol Levels and Intraocular Pressure in Healthy Adult Subjects. *Frontiers in Medicine, 8,* 736792. https://doi.org/10.3389/fmed.2021.736792 11. Scheidweiler, K. B., Himes, S. K., Desrosiers, N. A., & Huestis, M. A. (2016). In vitro stability of free and glucuronidated cannabinoids in blood and plasma collected in plastic gray-top sodium fluoride tubes following controlled smoked cannabis. *Forensic Toxicology, 34*(1), 179–185. https://doi.org/10.1007/s11419-015-0290-9 12. Schwope, D. M., Karschner, E. L., Gorelick, D. A., & Huestis, M. A. (2011). Identification of recent cannabis use: Whole-blood and plasma free and glucuronidated cannabinoid pharmacokinetics following controlled smoked cannabis administration. *Clinical* *Chemistry, 57*(10), 1406–1414. https://doi.org/10.1373/clinchem.2011.171777 13. Smith, R. C., Sershen, H., Janowsky, D. S., Lajtha, A., Grieco, M., Gangoiti, J. A., Gertsman, I., Johnson, W. S., Marcotte, T. D., & Davis, J. M. (2022). Changes in Expression of DNA-Methyltransferase and Cannabinoid Receptor mRNAs in Blood Lymphocytes After Acute Cannabis Smoking. *Frontiers in Psychiatry, 13,* 887700. <https://doi.org/10.3389/fpsyt.2022.887700> 14. Vandrey, R., Umbricht, A., & Strain, E. C. (2011). Increased Blood Pressure After Abrupt Cessation of Daily Cannabis Use. *Journal of Addiction Medicine, 5*(1), 16–20. https://doi.org/10.1097/ADM.0b013e3181d2b309 |
| Controlled smoking (n = 6) | 1. Bellville, J. W., Gasser, J. C., Miyake, T., & Aqleh, K. (1976). Tolerance to the respiratory effects of marijuana in man. *The Journal of Pharmacology and Experimental Therapeutics, 197*(2), 326–331. 2. Kelly, T. H., Foltin, R. W., Mayr, M. T., & Fischman, M. W. (1994). Effects of delta 9-tetrahydrocannabinol and social context on marijuana self-administration by humans. *Pharmacology, Biochemistry, and Behavior, 49*(3), 763–768. https://doi.org/10.1016/0091-3057(94)90099-x 3. Marsot, A., Audebert, C., Attolini, L., Lacarelle, B., Micallef, J., & Blin, O. (2017). Population pharmacokinetics model of THC used by pulmonary route in occasional cannabis smokers. *Journal of Pharmacological and Toxicological Methods, 85*, 49–54. https://doi.org/10.1016/j.vascn.2017.02.003 4. Tashkin, D. P., Gliederer, F., Rose, J., Chang, P., Hui, K. K., Yu, J. L., & Wu, T. C. (1991). Tar, CO and delta 9THC delivery from the 1st and 2nd halves of a marijuana cigarette. *Pharmacology, Biochemistry, and Behavior, 40*(3), 657–661. <https://doi.org/10.1016/0091-3057(91)90378-f> 5. Chao, T., Radoncic, V., Hien, D., Bedi, G., & Haney, M. (2018). Stress responding in cannabis smokers as a function of trauma exposure, sex, and relapse in the human laboratory*. Drug and Alcohol Dependence, 185*, 23–32. https://doi.org/10.1016/j.drugalcdep.2017.11.021 6. Urban, N., Martinez, D., Wang, Z., Grassetti, A., Wang, D., & Haney, M. (2015). Imaging the effect of deep rTMS on brain activity in chronic cannabis use. https://doi.org/10.1038/npp.2015.327 |
| Other drug prior CSA (n = 2) | 1. Lee, D., Vandrey, R., Mendu, D. R., Murray, J. A., Barnes, A. J., & Huestis, M. A. (2015). Oral fluid cannabinoids in chronic frequent cannabis smokers during ad libitum cannabis smoking. *Drug Testing and Analysis, 7*(6), 494–501. <https://doi.org/10.1002/dta.1718> 2. Lee, D., Bergamaschi, M. M., Milman, G., Barnes, A. J., Queiroz, R. H. C., Vandrey, R., & Huestis, M. A. (2015). Plasma Cannabinoid Pharmacokinetics After Controlled Smoking and Ad libitum Cannabis Smoking in Chronic Frequent Users. Journal of Analytical Toxicology, 39(8), 580–587. https://doi.org/10.1093/jat/bkv082 |
| Other administration (n = 2) | 1. Bosnyak, D., McDonald, A. C., Gasperin Haaz, I., Qi, W., Crowley, D. C., Guthrie, N., & Evans, M. (2022). Use of a Novel EEG-Based Objective Test, the Cognalyzer®, in Quantifying the Strength and Determining the Action Time of Cannabis Psychoactive Effects and Factors that May Influence Them Within an Observational Study Framework. *Neurology and Therapy, 11*(1), 51–72. https://doi.org/10.1007/s40120-021-00293-w 2. Wurz, G. T., Montoya, E., & DeGregorio, M. W. (2022). Examining impairment and kinetic patterns associated with recent use of hemp-derived Δ8-tetrahydrocannabinol: Case studies. *Journal of Cannabis Research, 4*(1), 36. https://doi.org/10.1186/s42238-022-00146-9 |
| Other paradigms (n = 4) | 1. Babor, T. F., Lex, B. W., Mendelson, J. H., & Mello, N. K. (1984). Marijuana, affect and tolerance: A study of subchronic self-administration in women. *NIDA Research Monograph*, *49*, 199–204. 2. Babor, T. F., Mendelson, J. H., Greenberg, I., & Kuehnle, J. C. (1975). Marijuana Consumption and Tolerance to Physiological and Subjective Effects. *Archives of General Psychiatry*, *32*(12), 1548. https://doi.org/10.1001/archpsyc.1975.01760300086007 3. Babor, T. F., Mendelson, J. H., & Kuehnle, J. (1976). Marihuana and human physical activity. *Psychopharmacology*, *50*(1), 11–19. https://doi.org/10.1007/BF00634148 4. Babor, T. F., Mendelson, J. H., Uhly, B., & Kuehnle, J. C. (1978). Social Effects of Marijuana Use in a Recreational Setting. *International Journal of the Addictions*, *13*(6), 947–959. https://doi.org/10.3109/10826087809039315 |
| Placebo Cannabis only (n = 1) | 1. Chait, L. D., & Perry, J. L. (1992). Factors influencing self-administration of, and subjective response to, placebo marijuana: *Behavioural Pharmacology*, *3*(6), 545???552. https://doi.org/10.1097/00008877-199212000-00002 |
| Abstract only (n = 2) | 1. Kosnett, M., Brooks-Russell, A., SWang, G., Dooley, G., Milavetz, G., Friedman, K., Heard, S., Thibodeaux, C., & Schwarz, J. (2020). Blood cannabinoid molar metabolite ratio is superior to blood THC as an indicator of recent cannabis smoking. *ACMT 2020 Annual Scientific Meeting, 16*, 133–134. https://doi.org/10.1007/s13181-020-00759-7 2. Morgan, C., Schafer, G., Gardener, C., & Curran, H. (2011). CBD vs. THC : How do effects differ in users prone to psychosis, acutely and chronically, and naturalistically and in the laboratory? *Abstracts for the 13th International Congress on Schizophrenia Research (ICOSR), 37.* https://doi.org/10.1093/schbul/sbq173 |

**Supplementary Table 3. Instructions Used for Ad Libitum smoking**

| **Reference** | **Ad libitum Instruction and Procedures** |
| --- | --- |
| ^a^ (Brands et al., 2019; Matheson, Mann, et al., 2020; Matheson, Sproule, et al., 2020) | “Please smoke the cigarette as you normally do when you smoke cannabis.  Please only smoke as much as you need to in order to experience the same ‘high’ that you are used to experiencing regularly.  You DO NOT need to smoke the whole cigarette, and you are free to stop smoking at ANY time and for ANY reason.  If you feel unwell, strange, or sick please STOP smoking and signal for help. Though no one will be in the room with you while you are smoking, you will be monitored from the next room for your safety.” |
| (Cappell et al., 1973) | “…subjects were told that they were to self-administer marihuana until they had attained a subjectively determined “optimal” high”… Cigarettes were made available one at a time upon demand. It was stressed that subjects were to smoke at their own rate and were not to consume any more than necessary to achieve the task, even if this meant discarding most of a cigarette.” |
| (Chait, 1989) | “Subjects were informed that they could smoke as much or as little marijuana as they wanted during the designated 30-min smoking period. They were also told that they should come to sessions wanting and expecting to ‘get high’ and that if there was a good reason why they would not want to (such as a final examination the following morning), they were advised not to attend that evening and to reschedule that session.” |
| ^a^ (Heishman et al., 1989) | “Subjects…smoked ad lib a single marijuana cigarette, they were stopped after the eighth puff was exhaled.”  Other than the standardized instructions given at the start of the puff inhalation, participants could smoke each of the 8 puffs however they liked. |
| (Herning et al., 1986) | “They were asked to smoke as they usually did, except the cigarette had to be smoked down to a mark 8 mm from the butt end. The experimenter-specified butt length was to require subjects to smoke the same amount of marijuana, thus maximizing opportunity for smoking behavior differences.” |
| ^a^ (Herrmann et al., 2015) | Participants could smoke as much or as little as they wanted within the 1 hour time limit. |
| (Hoffman et al., 2021; Marcotte et al., | "Smoke the cigarette the way you do at home to get high. You may take up to 10 minutes.” |
| (Matthias et al., 1997) | “Subjects were asked to smoke each marijuana cigarette ad lib but were specifically instructed to stop smoking once they had achieved their desired level of intoxication (’high’).” |
| ^a^ (McClure et al., 2012) | No instructions were given with regard to amount, duration, or manner of smoking. |
| (Meyer et al., 1971) | “…the subject was told to smoke all of the marijuana in a small pipe that was provided. The subject was allowed to smoke as he normally would but efforts were made to see that each dose of marijuana in the pipe was completely burned before the pipe was refilled. Instructions for the ad lib dose were slightly different. Here the subject was told that he could smoke as much marijuana as he wished and he was asked to try to get as ‘high’ as he usually got when using marijuana.” |
| (Miller & Cornett, 1978) | “They were allowed to smoke in any manner as they desired but were instructed to finish as much of the butt as possible. Smoking lasted about 10 min with all testing being completed in a quiet comfortable room.” |
| (Miller, Cornett, Brightwell, McFarland, Drew, et al., 1977) | “Subjects were allowed to smoke in any manner as they desired, but were instructed to finish as much of the cigarette as possible. Smoking took approximately 10 min and all testing was completed in a quiet, comfortable room.” |
| (Miller, Cornett, Drew, McFarland, Brightwell, et al., 1977) | “Subjects were allowed to smoke in any manner they desired, but were instructed to finish as much of the butt as possible. Smoking took approximately 10 min and all testing was completed in a quiet, comfortable room ” |
| (Miller et al., 1978) | “They were allowed to smoke in any manner they desired but were instructed to finish as much of the butt as possible. Smoking took about 7 and 10 min.” |
| (Miller et al., 1979) | “Subjects… were allowed to smoke in any manner they wished but were instructed to finish as much of the cigarette as possible. Smoking lasted 10-15 minutes with all testing being completed in a quiet comfortable room.” |
| (Miller, McFarland, Cornett & Brightwell, 1977) | “Subjects were allowed to smoke in any manner they desired, but were instructed to finish as much of the butt as possible. Smoking took approximately 10 min and all testing was completed in a quiet, comfortable room.” |
| (Miller, McFarland, Cornett, Brightwell, et al., 1977) | “The subjects were allowed to smoke in any manner they wished but were instructed to consume as much of the cigarette as possible… Smoking lasted approximately 10 min.” |
| (Perez-Reyes et al., 1981) | “The subjects were instructed to smoke at their customary rate and to finish the cigarettes completely.” |
| (Perez-Reyes et al., 1982) | “Subjects were instructed to smoke the cigarettes in their customary fashion and to smoke until they had reached what they had estimated to be their usual level of ‘high’ or for as long as they wished.” |
| (Schaefer et al., 1977) | “…subject was asked to smoke completely, using a ‘roach clip’, according to his own smoking technique.” |
| (Schwope et al., 2012) | “Participants smoked a single cigarette *ad libitum* for 10 min while seated with legs elevated, and remained in this position during the data collection period.” |
| ^a^ (Spindle et al., 2018, 2019) | Participants could smoke *ad libitum* until all the cannabis in the pipe was consumed. The study was designed to deliver a certain amount of THC within a specific time period. |
| ^a^ (Spindle et al., 2021) | Participants could smoke *ad libitum* until all the cannabis in the pipe was consumed. The study was designed to deliver a certain amount of THC within a specific time period. |
| (Tashkin et al., 1976) | “Each day thereafter for the following 80 days, except for one week between the 76^th^ and 82 days in the hospital, subjects smoked as many marihuana cigarette as desired…” |
| (Wu et al., 1988) | “Subjects were asked to smoke each joint in a manner as similar as possible to their usual pattern of smoking.” |
| (Zacny & De Wit, 1991) | “For the next 30 min. you can smoke more of the same kind of marijuana that you smoked earlier this evening. Whether you smoke or how much you smoke is entirely up to you.” |
| ^a^ Information received from author about how participants administered cannabis *ad libitum* | |
